# A randomized factorial trial of internet-delivered cognitive behavioural therapy: An 8-week program with or without extended support and booster lesson

**DOI:** 10.1016/j.invent.2022.100499

**Published:** 2022-02-06

**Authors:** H.D. Hadjistavropoulos, V. Peynenburg, D.L. Thiessen, M. Nugent, E. Karin, B.F. Dear, N. Titov

**Affiliations:** a 3737 Wascana Parkway, Department of Psychology, University of Regina, Regina, SK S4S 0A2, Canada; b 3737 Wascana Parkway, Department of Mathematics & Statistics, University of Regina, Regina, SK S4S 0A2, Canada; c eCentreClinic, Department of Psychology, Macquarie University, Sydney, NSW 2109, Australia; d MindSpot Clinic, Australian Hearing Hub Building, eCentreClinic, Department of Psychology, Macquarie University, Sydney, NSW 2109, Australia

**Keywords:** Internet-delivered, Cognitive Behaviour Therapy, Therapist support, Treatment duration, Booster sessions

## Abstract

While internet-delivered cognitive behavioural therapy (ICBT) is effective, some patients suggest extended support post-treatment could improve care. In this randomized factorial trial, we examined the benefits of an 8-week therapist-assisted ICBT program offered with or without an optional 4-week extension of support (Factor 1) and with or without an optional booster lesson (Factor 2). Patients screened for ICBT for depression and/or anxiety were randomly assigned to the conditions (*N* = 434) and we examined the use of the extension and booster, differences between those who did or did not use extension or booster, and the impact of the extension or booster on outcomes, engagement, and satisfaction at 26-weeks post-enrollment. Therapists recorded time and observations with offering support during the extension and booster. In the extension group, 54.4% (*n* = 56) requested the extension, while in the booster group 50.9% (*n* = 56) accessed the booster, and in the combined group, 41.6% (*n* = 47) requested the extension and 51.3% (*n* = 58) accessed the booster. Those who requested the extension were older, and more likely to report medication and mental health service use and severe mental health-related disability at pre-treatment; they also reported putting less effort into ICBT and finding skills more difficult. The booster was more often used among those with lower symptom severity, and those who put more effort into and had more positive experiences with ICBT. As expected, those assigned to extension sent more messages to their therapist, and those assigned to booster logged in more often. Therapists also took more time to deliver ICBT with an extension (>18 min) or booster (>13 min) compared to the 8-week program, and perceived extension and booster as beneficial for some, but not all patients. Treatment satisfaction was high across conditions, and effect sizes were large from pre-treatment to 26-week follow-up on most measures. No significant group differences were found in this study. Lack of group differences, however, could reflect low use of the extension and booster. Results provide helpful information about the demand for extensions and boosters, and provide directions for future research.

## Highlights

• Randomized controlled factorial trial of ICBT in routine care
• Examined extended support (yes/no) and booster lesson (yes/no)
• Use of the extension and booster was observed in roughly half of patients
• Extension use greater among those with greater severity and disability
• Booster use greater with lower symptom severity and positive treatment experiences

## 1 Introduction

There is extensive research demonstrating that internet-delivered cognitive behavioural therapy (ICBT), especially when accompanied by therapist support, results in significant improvements for mental health symptoms, including depression, generalized anxiety, panic, social anxiety, and posttraumatic stress (e.g., Andersson et al., 2019; Baumeister et al., 2014; Carlbring et al., 2018; Etzelmueller et al., 2020; Karyotaki et al., 2021; Romijn et al., 2019). In ICBT, patients learn various cognitive behavioural strategies by reviewing weekly online lessons. In routine care, therapist support most often accompanies these lessons in the form of weekly phone calls and or emails over an 8-week period (Etzelmueller et al., 2020).

In routine care, there is interest in identifying factors that may improve ICBT engagement and outcomes (Titov et al., 2018, Titov et al., 2019). One suggestion offered by patients is to extend the length of support (Hadjistavropoulos et al., 2018a) to allow patients greater time to work on skills and overcome barriers that can interfere with patient completion of ICBT within pre-specified timelines. While patients have made this suggestion, no study has explored whether and which patients would use or benefit from extended support. In general, offering extended support would be consistent with past research showing that patients' treatment preferences affect attrition, adherence, satisfaction, and outcomes (Preference Collaborative Review Group, 2008). On the other hand, however, there is correlational research showing that poorer outcomes are associated with offering patients “deadline flexibility” in completion of ICBT (Paxling et al., 2013). If an extension is similar to deadline flexibility, it is possible that an extension may be detrimental. Nevertheless, it needs to be emphasized that past research on deadline flexibility and outcomes is correlational (while deadline flexibility could lead to poor outcomes, it is also possible that those asking for deadline flexibility do so because they are not benefitting from ICBT). Ultimately, it is important to test the impact of an extension on ICBT outcomes.

While patients often suggest an extension as a strategy to improve ICBT, another potential method of addressing patient preference for extended support would be to offer a booster session (content and support) at a follow-up period. Booster sessions in face-to-face therapy typically consist of following up with patients post-treatment, offering a summary of symptom reduction strategies covered during treatment, combined with some support during this period (Baker and Wilson, 1985; Whisman, 1990). Booster sessions are regarded as beneficial either for improving or maintaining treatment outcomes by motivating patients to continue to practice skills taught during treatment, or by offering patients an opportunity to further enhance treatment skills (Whisman, 1990). In a meta-analysis of booster sessions among adults who received face-to-face therapy, Whisman (1990) found booster sessions were moderately successful across various treatment programs for various disorders. Specifically, Whisman (1990) observed booster sessions were effective at maintaining symptom improvements (e.g., in depression) and behavioural change (e.g., reduced smoking, weight loss) in 58% of the studies analyzed. Whisman (1990) concluded that although booster sessions do not necessarily prevent relapse of symptoms, they appear helpful in maintaining treatment gains over a longer period than would otherwise be expected. Furthermore, if patients begin to experience a recurrence of symptoms following treatment, booster sessions appear as effective as restarting the entire treatment program (Whisman, 1990). An important finding related to booster sessions is that the ability of a booster session to prevent relapse partially depends on how successful the initial intervention was, with booster sessions appearing ineffective when the treatment was initially unsuccessful at reducing symptoms (Baker and Wilson, 1985).

In terms of ICBT, there is limited research on booster sessions for the treatment of depression or anxiety. One study, however, explored the effects of three booster sessions that provided a summary of treatment content combined with therapist support offered at six months following ICBT for obsessive-compulsive disorder (Andersson et al., 2014). Results were promising and indicated booster sessions resulted in fewer relapses and improved general functioning compared to the ICBT without booster sessions at 7, 12, and 24-months post-treatment.

The purpose of the current factorial randomized controlled trial study was to advance understanding of how to and whether to extend ICBT in routine care. We were specifically interested in: 1) whether patients would request and use an extension of therapist support from 8 weeks up to 12 weeks; 2) whether patients would use a booster lesson offered at 16 weeks post-enrollment; 3) whether patients who used the extension and booster differed from those who did not; and 4) whether the extension, the booster, or the combination of these options would improve outcomes (primary outcomes were depression and generalized anxiety) over the 8 week ICBT program alone from pre-treatment to 26-weeks post-enrollment, patient engagement with ICBT assessed at 26-week follow-up (e.g., lessons completed, emails sent to therapist, log-ins), and treatment experiences with ICBT rated at 26-week follow-up (e.g., treatment satisfaction, negative effects). In accordance with implementation research (Hermes et al., 2019), therapists recorded the time required to offer support and documented experiences with offering the extension, the booster, or the combination. No hypotheses were formulated regarding how often and who would use the extension or the booster. It was hypothesized that outcomes, usage, and satisfaction would be stronger when patients were offered an extension, the booster, or the combination compared to the 8-week therapist-assisted ICBT program alone. The extension and booster were expected to increase therapist time, although the extent of the increase was not predicted.

## 2 Methods

### 2.1 Design and ethics

This study was a pragmatic 2 × 2 factorial randomized trial. Factorial trials represent a preferred approach when seeking to optimize a treatment (Collins et al., 2005) as the design permits examination of interventions under varying conditions and the opportunity to examine interactions without significantly increasing sample sizes (Kahan et al., 2020). The two factors included were: extension or not (factor 1) and booster or not (factor 2). Therapists and patients could not be blinded to condition given the nature of the factors studied. After obtaining research ethics board approval from the University of Regina, the trial was registered (ClinicalTrials.gov NCT04228575). Assuming power of 80% and alpha of 0.10 (as recommended by Collins et al., 2005 when optimizing treatment), a total sample size of 397 participants was calculated as sufficient to detect *d* = 0.25 between group effects and two-way interactions. To allow for the fact that some patients do not start ICBT in routine care (e.g., Hadjistavropoulos et al., 2020b), we recruited 469 patients (18% increase).

### 2.2 Patient recruitment, screening, and randomization

Recruitment took place between January 24th and October 6th, 2020. To begin, interested patients visited the Online Therapy Unit website (www.onlinetherapyuser.ca) and completed an online consent form and online screening questionnaire followed by a brief telephone interview that was primarily designed to confirm inclusion and exclusion criteria and ensure patient understanding of ICBT. Patients were considered eligible if they: 1) were 18 or older; 2) endorsed symptoms of depression and/or anxiety (diagnosis was not required); 3) were Saskatchewan residents and would be in the province for at least 8 weeks; 4) reported access to a computer and the Internet; 5) provided a medical contact for emergency purposes; and 6) had interest in and consented to ICBT. Exclusion criteria included: 1) recent hospitalization and high risk of suicide; 2) severe alcohol or drug problems; 3) weekly mental health treatment; 4) seeking help for a different mental health condition; and 5) self-reported medical condition that the patient anticipated would interfere with participation.

**Fig. 1:**
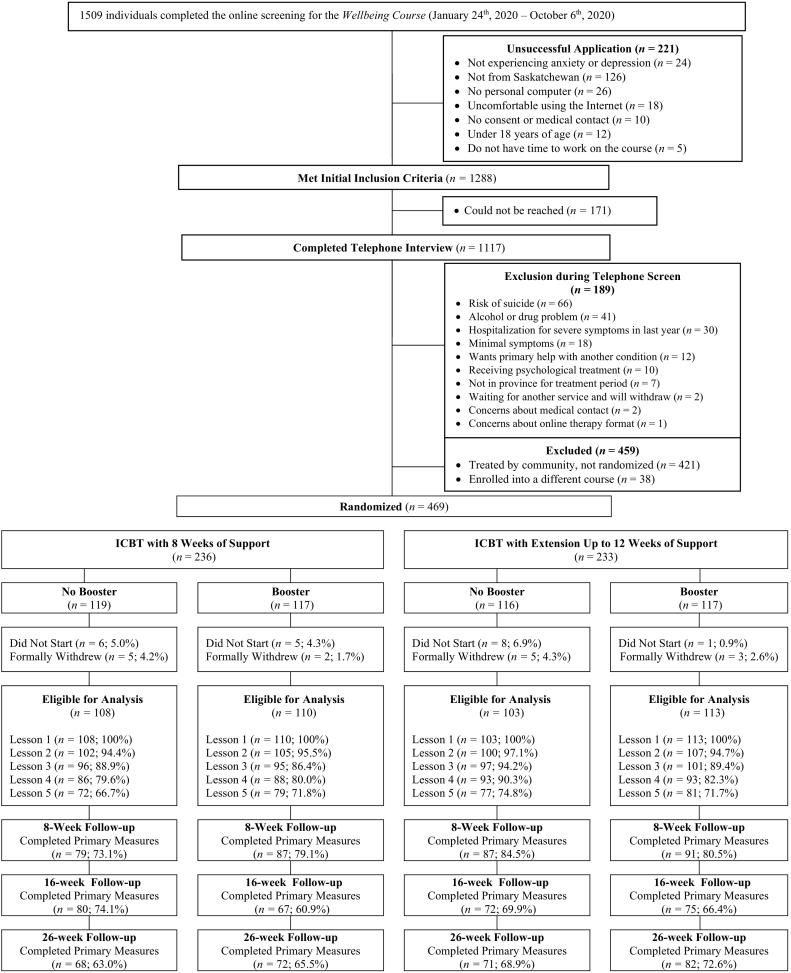
Patient flow from screening to 26-week follow-up.

The Online Therapy Unit is based at the University of Regina and is funded by the provincial government to deliver ICBT to residents throughout Saskatchewan at no cost to patients. The Unit screens patients for ICBT, and then on a 1-to-1 ratio either delivers ICBT to patients or assigns patients to receive ICBT provided by therapists employed by a community mental health clinic (see Hadjistavropoulos et al., 2016a).^1^ In this study, all eligible patients who were allocated to therapists working for the Online Therapy Unit (*n* = 469) were part of the trial as this unit received separate funding to support this research. Immediately after being allocated to therapists working in the Online Therapy Unit, screeners used Research Electronic Data Capture (REDCap) to randomly assign patients meeting the above conditions into 1 of 4 unique conditions created by the 2 × 2 design: ICBT, ICBT-extension, ICBT-booster, or ICBT-extension + booster. The computer-generated, permuted block randomization, with a fixed block size of 8, created a 1:1:1:1 allocation ratio. See Fig. 1 for patient flow.

### 2.3 Intervention

All patients received access to the *Wellbeing Course* developed by the eCentreClinic at Macquarie University and licensed by the Online Therapy Unit (for further details see Titov et al., 2015). This course consists of 5 core lessons covering: 1) the cognitive behavioural model of anxiety and depression; 2) thought challenging; 3) controlled breathing and activity scheduling; 4) graded exposure; and 5) relapse prevention planning and goal setting. Each lesson consists of 50 to 70 presentation-like slides, a downloadable guide that includes recommended homework assignments related to the core skills, frequently asked questions, and patient stories. Additional resources (i.e., assertiveness, communication skills, managing beliefs, managing worry, mental skills, panic, posttraumatic stress disorder, sleep, postpartum depression/anxiety, COVID-19, emergency information) are accessible to patients at any time. The 5 lessons are released gradually over 8 weeks once patients complete the proceeding lesson, with lesson 1 available immediately, lesson 2 at start of week 2, lesson 3 at start of week 4, lesson 4 at start of week 5, and lesson 5 at start of week 7. Automated emails notify patients about lesson availability and content. On a weekly basis, patients are asked to complete symptom measures and answer open and closed-ended reflection questions about their use of skills.

All patients who started lesson 1 received once-weekly therapist support on a pre-determined day each week using a secure email system on the intervention platform (for research supporting once-weekly support see Hadjistavropoulos et al., 2017, Hadjistavropoulos et al., 2020, Hadjistavropoulos et al., 2020). All therapists who offered therapist support received training in ICBT (Hadjistavropoulos et al., 2012) and regular supervision and auditing (Hadjistavropoulos et al., 2018, Hadjistavropoulos et al., 2019, Hadjistavropoulos et al., 2020). Therapists were instructed to spend approximately 15 min per patient each week, and to base their emails to patients on patient progress on the completion of lessons, any completed questionnaires, and emails from patients. When clinically indicated (e.g., suicide risk, increase in symptoms), therapists had the flexibility to increase time spent on emails or phone patients (i.e., when symptoms increase by five or more points, patients endorsed frequent suicidal thoughts, patients had not logged in for a week, patient questions and concerns). In emails, therapists were instructed to: 1) show warmth and concern; 2) provide feedback on any new completed questionnaires; 3) highlight relevant lesson content; 4) address patient questions about skill acquisition or challenges; 5) reinforce progress and practicing skills; 6) manage any risks (e.g., suicide); and 7) remind patients of ICBT procedures as needed (e.g., timelines, next check-in).

### 2.4 Treatment conditions

#### 2.4.1 Level one: ICBT-extension

Patients in the ICBT-extension condition were presented with the following instructions at the beginning of week 6 when patients logged into the intervention platform:

As part of the *Wellbeing Course* you have the option of extending the course by an additional 4 weeks (up to 12 weeks of support). The intent behind the additional 4 weeks is to give you extra time for lesson review, skill practice, and offer additional support while you continue to work on lesson materials and the additional resources. During the 4-week extension, we ask that you have a specific goal in mind as to what you would like to accomplish during this extension. During the 4-week extension your online therapist will continue to contact you weekly for support in reaching your goal.

Patients were then asked: “Would you like a 4-week extension so you are provided with support for a total of 12 weeks instead of 8 weeks?”, and, if yes, “What is your goal for the 4-week extension?” where they could select one or more of the following options, “complete all lessons”, “practice skills”, “review additional resources” or “other”. Patients had up to two weeks to complete these questions. If patients didn't request the extension, therapists concluded ICBT support at 8 weeks post-enrollment as usual. If patients requested the extension, therapists then concluded ICBT support at 12 weeks post-enrollment.

#### 2.4.2 Level two: ICBT-booster

At the end of the planned treatment period (8 weeks or 12 weeks if patients were assigned to and then selected extension), therapists informed patients assigned to the booster that at 16 weeks post-enrollment they would have access to an optional booster lesson (online materials that review core skills such as thought challenging, deep breathing, behavioural activation, and graded exposure) and an additional two weeks of therapist support. At 16 weeks, patients were subsequently sent the following automated email reminder to log in for the booster lesson:

In the Booster Lesson, we will review key concepts and skills from the *Wellbeing Course*. We will also discuss how to maintain motivation and continue to practice your skills regularly. The Booster Lesson comes with a Do-It-Yourself Guide so you can print and practice the skills you learned throughout the course without being online. You will have access to your therapist during the two-week Booster Lesson to ask any questions you have. Your therapist will email through the website shortly with more details.

At 16-weeks, the therapist then sent a personalized message to the patient on the intervention platform indicating that they would be available over the next two weeks should the patient require support. Subsequent therapist contact only took place if patients emailed therapists.

### 2.5 Measures

Unless otherwise specified, all measures listed below were administered at pre-treatment screening, and at 8-, 16-, and 26-weeks after randomization. To assist with provision of therapist support, primary outcome measures were also administered on a weekly basis starting at week 2 until the support period ended (week 8 for ICBT, week 12 for ICBT-extension). If patients completed the booster, patients were also administered primary outcome measures at week 16 and week 17 to allow therapists to review symptoms.

#### 2.5.1 Primary outcomes

##### 2.5.1.1 Patient Health Questionnaire (PHQ-9)

The PHQ-9 measures depression with 9 items creating a total score ranging from 0 to 27 (Kroenke et al., 2001, Kroenke et al., 2010). A PHQ-9 score ≥ 10 is commonly used to identify those with probable major depressive disorder (Manea et al., 2012). Cronbach's alpha in this study ranged from 0.82 to 0.88.

##### 2.5.1.2 Generalized Anxiety Disorder-7 (GAD-7)

The GAD-7 measures anxiety with 7 items resulting in a total score ranging from 0 to 21 (Spitzer et al., 2006). A GAD-7 score ≥ 10 is commonly used to identify those likely meeting diagnostic criteria for generalized anxiety disorder (Spitzer et al., 2006). Cronbach's alpha in this study ranged from 0.86 to 0.91.

#### 2.5.2 Secondary outcomes^2^

##### 2.5.2.1 Sheehan Disability Scale (SDS)

The SDS measures functional impairment in work/school, social life, and family life by asking patients to rate items on 0 to 10 scale; total scores range from 0 to 30 (Sheehan, 1983). Cronbach's alpha in this study ranged from 0.78 to 0.90.

##### 2.5.2.2 Panic Disorder Severity Scale Self-Report (PDSS-SR)

The PDSS-SR assessed panic disorder symptoms with 7 items resulting in a total score ranging from 0 to 28 (Shear et al., 2001). A PDSS-SR score ≥ 8 is commonly used to identify those who are likely to have panic disorder (Allen et al., 2016). Cronbach's alpha in this study ranged from 0.88 to 0.91.

##### 2.5.2.3 Social Interaction Anxiety Scale and Social Phobia Scale (SIAS-6/SPS-6)

The SIAS-6 and SPS-6, consisting of 6 items each, measured social anxiety; items were summed to create a total score ranging from 0 to 48 (Peters et al., 2012). A SIAS-6 score of ≥7 and SPS-6 score ≥ 2 are often used to identify those likely to have social anxiety disorder (Peters et al., 2012). Cronbach's α in this study ranged from 0.84 to 0.87 on the SIAS-6 and 0.91 to 0.93 on the SPS-6.

##### 2.5.2.4 Life Events Checklist for DSM-5 (LEC-5)

At screening, patients completed the LEC-5 to assess exposure to various potentially traumatic experiences (Weathers et al., 2013a) and those endorsing more than one event were asked to select the event causing the most distress.

##### 2.5.2.5 Posttraumatic Stress Disorder Checklist for DSM-5 (PCL-5)

Patients who endorsed a distressing traumatic event on the LEC at screening were subsequently administered the PCL-5 to assess symptoms of posttraumatic stress disorder. The measure consists or 20 items and total scores range from 0 to 80 (Weathers et al., 2013b). A score ≥ 33 is commonly used to identify those with a likely diagnosis of PTSD (Weathers et al., 2013b). Cronbach's α in this study ranged from 0.93 to 0.96.

##### 2.5.2.6 The EQ-5D-5L

Patients completed the EQ-5D-5L which assesses quality of life and general health (Herdman et al., 2011). Patients rated mobility, self-care, usual activities, pain/discomfort, and anxiety/depression on five-point Likert scales. They also rated their overall general health on a visual analogue scale (VAS) from 0 (worst health) to 100 (best health). The VAS is reported in this paper. The EQ-5D-5L will be analyzed in the future if we undertake an economic evaluation (Camacho et al., 2018).

##### 2.5.2.7 Mental health service use

At screening, patients answered questions about any lifetime use of the following providers or services for mental health reasons: family doctor/walk-in clinic, psychiatrist, psychologist, social worker, counsellor, nurse/community nurse/psychiatric nurse, occupational therapist, medical specialist, other health care professional, psychiatric day/part-time treatment program, alcohol or drug treatment program, self-help group, occupational stress injury program, hospital emergency room, ambulance/paramedics, crisis service, hospital admission. At screening and at 26-week follow-up, they were also asked for frequency of use of each of these same providers and services in the past three months.

##### 2.5.2.8 Medication use

At screening, patients were asked if they had ever taken medication for mental health concerns. At screening and at 26-week follow-up, they were then asked if they had taken medication in the past three months.

##### 2.5.2.9 Employment

At screening and 26-week follow-up, patients were asked about occupational status (e.g., paid work, run the household, retired, attending school, unfit for work because of emotional or physical reasons, or off work for other reasons). If patients selected that they had paid work, they were asked: 1) if mental health concerns kept them off work during the past three months; 2) to rate their job performance when at work on a scale from *1* (*much worse performance*) to *10* (*not affected*); and 3) whether they received disability benefits or workplace accommodations in the past three months.

#### 2.5.3 Treatment engagement

Treatment engagement was examined at 26-week follow-up in terms of number of: lessons accessed, days between first and last log in, logins, emails sent to therapist, emails from therapist to patient, and phone calls between patient and therapist.

#### 2.5.4 Treatment experiences

##### 2.5.4.1 Credibility and Expectancy Questionnaire (CEQ)

At pre-treatment, post-treatment, and 16- and 26-week follow-up, three items of the CEQ assessing treatment credibility were administered, which were summed to create a total score ranging from 3 to 27 (Devilly and Borkovec, 2000). Cronbach's α in this study ranged from 0.80 to 0.90.

##### 2.5.4.2 Treatment satisfaction

At 8-, 16- and 26-week follow-up, patients rated satisfaction with various aspects of treatment (i.e., overall treatment, website, quality of materials, knowledge and competence of therapist, phone calls (if applicable) and emails from therapists) on a “1-very dissatisfied” to “5-very satisfied” scale. They were also asked whether the treatment was worth their time (“Yes” or “No”) and whether they would recommend the treatment to a friend (“Yes” or “No”), and rated the extent to which the course affected their confidence in managing symptoms and their motivation to seek out future treatment if needed (rated “1-greatly reduced” to “5-greatly increased”).

##### 2.5.4.3 Negative effects

Negative effects or events associated with ICBT were assessed at 8-, 16- and 26-week follow-up. Patients were first asked whether they had experienced unwanted negative effects/events (“Yes” or “No”), which was followed by questions about the impact the negative effects/events, and whether the negative effects/events continue to have an impact (rated “0-no negative impact” to “3-severe negative impact”).

### 2.6 Therapist experiences

Therapists tracked total time required to deliver ICBT to each patient each week. At the end of the trial, therapists met and provided written comments on their positive and negative experiences with offering the extension, the booster, and the combination.

### 2.7 Data analysis

To describe the patient sample, descriptive statistics were conducted. Subsequently, we examined the proportion who used the extension and the booster and whether there were differences among those who made use of these conditions compared to those who did not. Comparisons were made using chi-square tests and one-way ANOVAs.

Multiple imputations were used to accommodate missing responses, using an approach consistent with other research on internet interventions (Karin et al., 2021). To increase the plausibility of a missing at random assumption (Little and Rubin, 2014) and following a recommendation to use an “inclusive” approach to creating imputation models (Collins et al., 2001), we specified imputation models controlling for any variables that were associated with outcome measures or response rates. For each outcome measure the imputation models controlled for that outcome measure at other observation times, treatment condition, interactions between treatment condition and pre-treatment outcome measure, and variables that were associated with response rates (i.e., age, gender, education level, employment status, ethnicity, number of lessons accessed by week 8, and pre-treatment PHQ-9, PDSS-SR, PCL-5). 50 imputations were created in R using the MICE package (van Buuren and Groothuis-Oushoorn, 2011) and predictive mean matching.

Generalized estimating equations (GEE) were used to estimate mean outcomes while accounting for within-subject correlation using robust error estimates (Hubbard et al., 2010; Liang and Zeger, 1986). A gamma distribution with log-link was used to accommodate skewed response distributions and model symptom changes as proportional to pre-treatment symptom severity (Karin et al., 2018). The GEE models were fit in R with the geepack package (Højsgaard et al., 2006; Yan and Fine, 2004; Yan, 2002) and used exchangeable working correlations. Hypothesis tests for the GEEs were performed using Wald tests pooling results from the multiple imputations.

To compare outcomes between treatment conditions we calculated marginal means, proportional changes from pre-treatment, and Cohen's *d* effect sizes along with 95% confidence intervals, focusing on results at week 26. As planned with the factorial design, we first checked for 3-way time ∗ extension ∗ booster interactions and then examined the effects of each factor. Given that not all patients requested the extension and not all used the booster, we also performed two sub-group analyses to examine whether actual usage of extension or booster would lead to further improvements. Specifically, for any patient offered extension, we compared changes from week 8 (when extension became available) to week 26 between patients who did or did not request extension. Secondly, for any patient offered the booster, we compared changes from week 16 (when the booster became available) to week 26 between patients who did or did not access the booster.

Analyses were also conducted to examine whether treatment engagement, treatment experiences and therapist time varied as a function of group and interactions between groups. Two-way ANOVAs were conducted to examine continuous outcome variables and factorial logistic regression analyses to examine categorical outcome variables. Throughout all analyses, alpha was set at 0.10 in order to ensure that trends in data were identified (as recommended by Collins et al., 2005 when optimizing treatment). Therapist observations regarding extension, the booster, or the combination were summarized based on therapist notes; this summary was then reviewed and revised by therapists to ensure the observations accurately described their experiences.

## 3 Results

### 3.1 Patient background

Patient background variables at pre-treatment are reported in Table 1. The mean age of patients was 35.90 years (*SD* = 12.10), 75.6% (*n* = 328) were women, 85.90% (*n* = 373) were White, 61.3% (*n* = 266) were married/common-law, only 18.0% (*n* = 78) reported high school education or less, 55.5% (*n* = 241) reported part- or full-time employment, and 56.2% (*n* = 244) reported living in a city > 100,000 residents. Most patients had utilized some type of mental health service at some point during their life (92.2%, *n* = 400), with family doctor/walk-in clinic/nurse/other health professional being the most common (88.5%, *n* = 384). At pre-treatment, the majority of patients reported using psychotropic medication (59.9%; *n* = 260) and had pre-treatment symptoms indicative of depression (76.7%; *n* = 333), generalized anxiety (74%; *n* = 321), and panic disorder (52.3%; *n* = 227). Additionally, 48.8% (*n* = 212) had scores suggestive of social anxiety and 30.9% (*n* = 134) had scores suggestive of posttraumatic stress disorder. On average, patients scored above clinical cut-offs on 2.83 (*SD* = 1.45) measures. Only 5.5% (*n* = 24) of patients did not have any pre-treatment scores within the clinical range.

**Table 1:** Pre-treatment patient characteristics by treatment condition.

Variable	All groups (*N* = 434)		ICBT (*n* = 108)		ICBT-extension (*n* = 103)		ICBT-booster (*n* = 110)		ICBT-extension + booster (*n* = 113)	
	*n*	%	*n*	%	*n*	%	*n*	%	*n*	%
Age										
Mean (*SD*)	35.90 (12.10)		34.54 (11.59)		34.85 (10.19)		37.27 (12.03)		36.83 (14.01)	
Range	18–79		18–70		18–61		20–71		18–79	

Gender										
Female	328	75.6	83	76.9	71	68.9	89	80.9	85	75.2
Male	101	23.3	23	21.3	31	30.1	19	17.3	28	24.8
Transgender	1	0.2	–	–	1	1.0	–	–	–	–
Non-binary	–	–	–	–	–	–	–	–	–	–
Two spirit	1	0.2	–	–	–	–	1	0.9	–	–
Prefer not to disclose	3	0.7	2	1.9	–	–	1	0.9	–	–

Marital status										
Single/dating/never married	118	27.2	29	26.9	33	32.0	22	20.0	34	30.1
Married/common-law	266	61.3	65	60.2	61	59.2	77	70.0	63	55.8
Separated/divorced/widowed	50	11.5	14	13.0	9	8.7	11	10.0	16	14.2

Education										
High school diploma or less	78	18.0	17	15.7	20	19.4	18	16.4	23	20.4
Some post-secondary	191	44.0	51	47.2	46	44.7	47	42.7	47	41.6
University degree	165	38.0	40	37.0	37	35.9	45	40.9	43	38.1

Employment status										
Employed part- or full- time	241	55.5	65	60.2	67	65.0	52	47.3	57	50.4
Unemployed	42	9.7	12	11.1	5	4.9	13	11.8	12	10.6
Homemaker	70	16.1	15	13.9	15	14.6	26	23.6	14	12.4
Student	41	9.4	13	12.0	9	8.7	8	7.3	11	9.7
Disability	53	12.2	11	10.2	11	10.7	12	10.9	19	16.8
Retired	14	3.2	3	2.8	1	1.0	2	1.8	8	7.1
Other reasons no paid work	59	13.6	14	13.0	9	8.7	19	17.3	17	15.0
Missed work in past 3 months due to mental health	120	27.6	23	21.3	33	32.0	31	28.2	33	29.2
Rating of job performance at work rated 0 to 10	5.35 (2.06)		5.38 (2.02)		5.32 (2.20)		5.62 (1.71)		5.06 (2.29)	

Ethnicity										
White	373	85.9	95	88.0	90	87.4	90	81.8	98	86.7
Indigenous	31	7.1	5	4.6	5	4.9	12	10.9	9	8.0
Other/prefer not to disclose	30	6.9	8	7.4	8	7.8	8	7.3	6	5.3

Location										
City over 100,000	244	56.2	68	63.0	57	55.3	58	52.7	61	54.0
Small city	44	10.1	12	11.1	9	8.7	11	10.0	12	10.6
Small rural (under 10,000)	146	33.6	28	25.9	37	35.9	41	37.3	40	35.4

Pre-treatment mental health characteristics										
Receiving treatment for mental health	198	45.6	43	39.8	47	45.6	49	44.5	59	52.2
Current use of psychotropic medication	260	59.9	64	59.3	65	59.1	58	56.3	73	64.2
Lifetime use of family doctor/walk-in/nurse/other health professional	384	88.5	86.1	93	90.0	99	89.3	92	88.5	100
Lifetime use of psychiatrist	153	35.3	30.6	33	43.6	48	28.2	29	38.1	43
Lifetime use of psychologist/counsellor/social worker	287	66.1	70.4	76	63.6	70	65.0	67	65.5	74
Lifetime use of any professional^a^	400	92.4	99	91.7	94	91.3	103	93.6	104	92.0
Lifetime treatment program use^b^	90	20.7	24	22.2	23	20.9	17	16.5	26	23.0
Lifetime use of emergency room/ambulance/crisis service	87	20.0	20	18.5	28	25.5	16	15.5	23	20.4
PHQ-9 ≥ 10	333	76.7	81	75.0	74	71.8	86	78.2	92	81.4
GAD-7 ≥ 10	321	74.0	78	72.2	76	73.8	80	72.7	87	77.0
PDSS-SR ≥ 8	227	52.3	56	51.9	59	57.3	50	45.5	62	54.9
SIAS ≥ 7 and SPS ≥ 2	212	48.8	50	46.3	60	58.3	47	42.7	55	48.7
PCL-5 ≥ 33	134	30.9	38	35.2	25	24.3	35	31.8	36	31.9
No scores in clinical range	24	5.5	5	4.6	9	8.7	5	4.5	5	4.4
Mean # scores > cut-off	2.83 (1.45)		2.81 (1.44)		2.85 (1.52)		2.71(1.38)		2.94 (1.47)	
Credibility of ICBT (3 to 27)	20.91 (4.47)		21.15 (4.37)		20.37 (4.26)		21.28 (4.46)		20.81 (4.77)	

Note. ICBT = Internet-delivered Cognitive Behaviour Therapy; PHQ-9 = Patient Health Questionnaire-9; GAD-7 = Generalized Anxiety Disorder-7; PDSS-SR = Panic Disorder Severity Scale Self- Report; SIAS = Social Interaction Anxiety Scale; SPS = Social Phobia Scale; PCL-5 = PTSD Checklist for DSM-5.

a Professionals include family doctor, walk-in clinics, nurse, other health professionals, psychiatrist, psychologist, counsellor, or social worker.

b Treatment programs include psychiatric day or part-time treatment programs, alcohol/drug programs, self-help groups, and occupational stress injury programs.

### 3.2 Use of extension and booster

In the ICBT-extension group, 54.4% (*n* = 56) requested extension, while in the ICBT-booster, 50.9% (*n* = 56) accessed the booster. Of those patients randomized to ICBT-extension + booster, 41.6% (*n* = 47) requested the extension and 52.2% (*n* = 59) used the booster, resulting in 26.5% (*n* = 30) having both extension and the booster, 15.0% (*n* = 17) extension only, 25.7% (*n* = 29) booster only, and 32.7% (*n* = 37) ICBT only. In terms of therapist contact, review of patient messages revealed that 35.0% (*n* = 36/103) who requested the extension never emailed their therapists during the extension, and 33.0% (*n* = 38/115) who accessed the booster similarly did not email their therapists during the booster.

Patients could check off multiple reasons for wanting an extension. The most common reason was to practice skills (91.3%; *n* = 94/103), followed by reviewing the additional resources (68.9%; *n* = 71/103), and completing all lessons from the *Wellbeing Course* (56.3%; *n* = 58/103). Five patients provided a response of ‘Other’, with two patients indicating that they wanted more therapist interaction, two patients indicating they wanted more time to complete the course, and one patient indicating a desire to specifically work on managing worry and sleep concerns.

### 3.3 Background differences among patients using extension and booster

Among those assigned to ICBT-extension, patients who requested the extension compared to those who did not were older (*M* = 38.35 years, *SD* = 13.08 vs *M* = 33.68 years, *SD* = 11.28; *F*_(1,212)_ = 7.81, *p* = .006), more likely to report mental health medication use in their lifetime (80.6% vs 68.0%; ꭙ^2^ (1, *N* = 213) = 4.16 *p* = .04), more likely to report some mental health treatment at pre-treatment (59.2% vs 39.8%; ꭙ^2^ (1, *N* = 216) = 8.12 *p* = .004), and to report more severe disability on the SDS at pre-treatment (*M* = 19.21, *SD* = 6.12 vs *M* = 17.46, *SD* = 6.99; *F*_(1,215)_ = 3.82, *p* = .05). Among those assigned to ICBT-booster, patients who actually accessed the booster compared to those who did not were older (*M* = 39.80 years, *SD* = 14.54 vs *M* = 34.16 years, *SD* = 10.60; *F*_(1,219)_ = 10.78, *p* = .001), had less severe scores at pre-treatment on the PHQ-9 (*M* = 13.13, *SD* = 5.33 vs *M* = 14.76, *SD* = 5.60; *F*_(1,221)_ = 4.91, *p* = .03), GAD-7 (*M* = 12.27, *SD* = 4.71 vs *M* = 13.58, *SD* = 5.00; *F*_(1,221)_ = 4.04, *p* = .05), PDSS-SR (*M* = 7.09, *SD* = 8.79 vs *M* = 9.51, *SD* = 6.69; *F*_(1,221)_ = 8.69, *p* = .004), and SPS-6 (*M* = 4.88, *SD* = 5.83 vs *M* = 7.73, *SD* = 7.38; *F*_(1,219)_ = 10.34, *p* = .002), and had fewer clinically significant pre-treatment scores overall (*M* = 2.59, *SD* = 1.37 vs *M* = 3.08 *SD* = 1.44; *F*_(1,221_ = 6.62, *p* = .01).

### 3.4 Primary outcomes

Table 2 presents the estimated means, standard deviations, percentage reductions, and effect sizes by factor (extension vs no extension; booster vs no booster). Supplementary Table 1 presents the same information for each treatment condition (ICBT, ICBT-extension, ICBT-booster, and ICBT-extension + booster). The predominant pattern that emerged from the GEE analyses was statistically significant time effects on both primary measures in all treatment conditions (*p* < .001). There were large reductions from pre-treatment to week 26 on the PHQ-9 (Cohen's *d*: 1.36–1.46) and GAD-7 (Cohen's *d*: 1.49–1.69). There was also a significant time ∗ extension ∗ booster interaction at week 26 on the GAD-7 (*p* = .07), but not on the PHQ-9 (*p* = .11). No time ∗ extension or time ∗ booster interactions were found at week 26 on either primary measure (*p* > .37). To follow-up on the 3-way interaction, pairwise comparisons on the GAD-7 showed that smaller improvement was found at week 26 for the ICBT booster + extension (54.7%) condition compared to ICBT-booster (64.1%, *p* = .09). Other differences were not significant.

**Table 2:** Estimated marginal means, standard deviations, 95% confidence intervals, percentage changes, and effect sizes (Cohen's *d*) for primary and secondary outcomes by treatment condition using pooled imputations.

	Estimated marginal means				Percentage changes from pre-treatment			Within-group effect sizes from pre-treatment	
	Pre-treatment	Post-treatment	16-Week follow-up	26-Week follow up	To post-treatment	To 26-week follow-up	*p*-Value^a^	To post-treatment	To 26-week follow-up
Primary outcomes									

PHQ-9									
No extension	13.48 (5.47)	6.88 (5.31)	6.23 (4.78)	5.94 (4.81)	49.0 [42.6, 55.4]	56.0 [49.9, 62.0]		1.22 [1.02, 1.43]	1.46 [1.25, 1.67]
Extension	13.82 (5.44)	7.06 (5.79)	6.38 (5.39)	6.34 (5.48)	48.9 [42.7, 55.1]	54.1 [48.2, 60.0]	0.64	1.20 [1.00, 1.41]	1.37 [1.16, 1.58]
No booster	13.36 (5.36)	7.24 (5.33)	6.31 (4.84)	6.25 (5.05)	45.8 [39.6, 52.0]	53.2 [47.1, 59.4]		1.14 [0.94, 1.35]	1.36 [1.15, 1.57]
Booster	13.93 (5.54)	6.71 (5.75)	6.30 (5.33)	6.04 (5.26)	51.8 [45.6, 58.1]	56.7 [51.0, 62.3]	0.37	1.28 [1.07, 1.48]	1.46 [1.25, 1.67]

GAD-7									
No extension	12.68 (4.83)	6.56 (5.09)	5.42 (4.34)	5.10 (4.12)	48.3 [42.0, 54.5]	59.8 [54.0, 65.5]		1.23 [1.03, 1.44]	1.69 [1.47, 1.90]
Extension	12.95 (5.14)	6.66 (5.51)	5.82 (5.27)	5.55 (4.78)	48.6 [42.3, 54.9]	57.1 [51.4, 62.8]	0.49	1.18 [0.97, 1.38]	1.49 [1.28, 1.70]
No booster	12.72 (5.09)	6.96 (5.31)	5.61 (4.71)	5.38 (4.45)	45.3 [38.7, 51.8]	57.7 [51.8, 63.5]		1.10 [0.90, 1.31]	1.53 [1.31, 1.75]
Booster	12.91 (4.89)	6.27 (5.28)	5.62 (4.94)	5.27 (4.48)	51.4 [45.3, 57.6]	59.2 [53.6, 64.8]	0.69	1.30 [1.10, 1.51]	1.63 [1.41, 1.84]

Secondary outcomes									

SDS									
No extension	17.96 (7.24)	12.25 (8.44)	8.85 (8.24)	7.97 (8.39)	31.8 [24.4, 39.3]	55.6 [48.2, 63.1]		0.73 [0.53, 0.92]	1.27 [1.07, 1.48]
Extension	18.30 (6.63)	10.87 (8.25)	8.98 (8.46)	7.71 (8.08)	40.6 [34.2, 47.0]	57.9 [50.8, 65.0]	0.63	0.99 [0.79, 1.19]	1.43 [1.22, 1.64]
No booster	17.80 (7.17)	12.12 (8.12)	9.16 (8.18)	7.08 (7.66)	31.9 [24.8, 39.1]	60.2 [53.1, 67.3]		0.74 [0.54, 0.94]	1.44 [1.23, 1.66]
Booster	18.44 (6.71)	11.03 (8.57)	8.68 (8.50)	8.56 (8.69)	40.2 [33.5, 46.9]	53.6 [46.3, 60.8]	0.15	0.96 [0.76, 1.16]	1.27 [1.07, 1.47]

PDSS-SR									
No extension	7.93 (6.21)	4.26 (4.51)	3.48 (4.35)	2.76 (3.63)	46.2 [36.4, 56.0]	65.2 [58.1, 72.3]		0.67 [0.48, 0.87]	1.01 [0.81, 1.21]
Extension	8.76 (6.22)	5.02 (4.86)	3.76 (4.76)	3.50 (4.17)	42.7 [34.2, 51.1]	60.0 [52.6, 67.5]	0.24	0.67 [0.47, 0.86]	0.99 [0.79, 1.19]
No booster	8.42 (6.21)	4.57 (4.75)	3.91 (4.97)	3.04 (3.98)	45.8 [36.6, 54.9]	63.9 [56.6, 71.3]		0.70 [0.50, 0.89]	1.03 [0.83, 1.23]
Booster	8.27 (6.25)	4.71 (4.66)	3.35 (4.13)	3.22 (3.87)	43.0 [33.8, 52.3]	61.1 [53.4, 68.9]	0.56	0.64 [0.45, 0.84]	0.97 [0.78, 1.17]

SIAS-6/SPS-6									
No extension	25.68 (10.32)	12.13 (10.41)	14.92 (12.11)	13.00 (11.88)	52.8 [46.6, 58.9]	49.4 [42.4, 56.3]		1.31 [1.10, 1.51]	1.14 [0.93, 1.34]
Extension	26.64 (10.11)	13.25 (10.37)	15.70 (12.65)	13.80 (11.67)	50.2 [44.5, 56.0]	48.2 [41.6, 54.8]	0.78	1.30 [1.10, 1.51]	1.17 [0.97, 1.38]
No booster	25.93 (10.19)	12.64 (10.42)	15.61 (12.13)	12.88 (11.17)	51.2 [45.2, 57.2]	50.3 [43.7, 56.9]		1.29 [1.08, 1.50]	1.22 [1.01, 1.43]
Booster	26.37 (10.26)	12.73 (10.39)	15.01 (12.61)	13.89 (12.32)	51.7 [45.8, 57.6]	47.3 [40.3, 54.3]	0.50	1.32 [1.11, 1.52]	1.10 [0.90, 1.30]

PCL-5									
No extension	34.70 (17.40)	25.09 (16.75)	17.62 (16.68)	14.72 (14.41)	27.7 [18.5, 36.9]	57.6 [49.4, 65.7]		0.56 [0.32, 0.80]	1.25 [0.99, 1.50]
Extension	32.29 (17.65)	24.27 (17.46)	18.06 (17.70)	15.05 (15.18)	24.8 [15.1, 34.6]	53.4 [44.8, 62.0]	0.43	0.46 [0.22, 0.70]	1.04 [0.79, 1.30]
No booster	32.41 (17.69)	23.53 (16.95)	17.40 (16.21)	13.97 (14.35)	27.4 [17.9, 36.8]	56.9 [48.3, 65.4]		0.51 [0.27, 0.75]	1.14 [0.88, 1.40]
Booster	34.57 (17.39)	25.79 (17.19)	18.26 (18.09)	15.76 (15.16)	25.4 [15.6, 35.2]	54.4 [46.1, 62.7]	0.64	0.51 [0.27, 0.74]	1.15 [0.90, 1.40]

EQ-VAS									
No extension	59.18 (19.39)	71.63 (20.16)	69.31 (16.98)	69.71 (16.91)	30.5 [22.6, 38.4]	25.8 [18.8, 32.8]		0.63 [0.44, 0.82]	0.58 [0.39, 0.77]
Extension	56.80 (19.69)	71.48 (19.44)	69.88 (16.71)	69.31 (18.27)	34.0 [27.0, 40.9]	29.0 [22.1, 35.8]	0.52	0.75 [0.55, 0.94]	0.66 [0.46, 0.85]
No booster	57.92 (18.87)	72.96 (17.55)	69.70 (16.81)	70.29 (17.05)	35.7 [29.0, 42.5]	29.4 [22.9, 35.9]		0.82 [0.63, 1.02]	0.69 [0.49, 0.88]
Booster	58.06 (20.22)	70.22 (21.64)	69.49 (16.89)	68.77 (18.07)	29.0 [20.8, 37.2]	25.5 [18.3, 32.8]	0.43	0.58 [0.39, 0.77]	0.56 [0.37, 0.75]

Note. ICBT = Internet-delivered Cognitive Behaviour Therapy; PHQ-9 = Patient Health Questionnaire-9; GAD-7 = Generalized Anxiety Disorder-7; SDS = Sheehan Disability Scale; PDSS-SR = Panic Disorder Severity Scale Self-Report; SIAS-6 = Social Interaction Anxiety Scale 6-item; SPS-6 = Social Phobia Scale 6-item; PCL-5 = PTSD Checklist for DSM-5; EQ VAS = EuroQol Visual Analogue Scale.

a Test of whether percentage changes from pre-treatment to week 26 are the same for both groups (no extension versus extension, no booster versus booster).

Table 3 compares patient outcomes by their actual usage of extension or booster. Patients who requested the extension did not have significantly different proportional changes from week 8 to week 26 on the PHQ-9 (*p* = .72) or GAD-7 (*p* = .43) compared to patients who did not request the extension. Patients who accessed the booster did not have significantly different proportional changes from week 16 to week 26 on the PHQ-9 (*p* = .32) or GAD-7 (*p* = .98) compared to patients who did not access the booster.

**Table 3:** Estimated marginal means, 95% confidence intervals, percentage changes, and effect sizes (Cohen's *d*) for primary and secondary outcomes based on if patient requested extension and accessed booster. Pooled results from multiple imputation.

	Estimated marginal means				Percentage change from post-treatment to 26-week follow-up	Within group effect size from post-treatment to 26-week follow-up	Percentage change from 16-week to 26-week follow-up	Within-group effect sizes from 16-week to 26-week follow-up
	Pre-treatment	Post-treatment	16-Week follow-up	26-Week follow up				
Primary outcomes								

PHQ-9								
Did not request extension	13.38 (5.23)	6.14 (5.59)	5.55 (4.79)	5.64 (4.86)	8.1 [−9.7, 25.9]	0.09 [−0.17, 0.36]	–	–
Requested extension	14.30 (5.66)	8.07 (5.86)	7.28 (5.86)	7.11 (6.01)	11.9 [−3.1, 26.9]	0.16 [−0.11, 0.43]	–	–
Did not access booster	14.76 (5.66)	7.49 (6.14)	6.73 (5.25)	6.80 (5.57)	–	–	−1.1 [−20.3, 18.1]	−0.01 [−0.28, 0.25]
Accessed booster	13.13 (5.33)	5.96 (5.25)	5.87 (5.37)	5.30 (4.85)	–	–	9.8 [−5.7, 25.3]	0.11 [−0.15, 0.37]

GAD-7								
Did not request extension	12.96 (4.95)	5.89 (4.92)	5.31 (4.71)	5.15 (4.31)	12.5 [−5.1, 30.1]	0.16 [−0.10, 0.42]	–	–
Requested extension	12.94 (5.36)	7.50 (6.00)	6.36 (5.78)	5.98 (5.21)	20.2 [6.2, 34.2]	0.27 [−0.01, 0.54]	–	–
Did not access booster	13.58 (5.00)	7.20 (5.70)	6.25 (5.12)	5.85 (4.82)	–	–	6.4 [−13.5, 26.3]	0.08 [−0.19, 0.35]
Accessed booster	12.27 (4.71)	5.37 (4.69)	5.01 (4.68)	4.70 (4.05)	–	–	6.1 [−9.5, 21.7]	0.07 [−0.19, 0.33]

Secondary outcomes								

SDS								
Did not request extension	17.46 (6.99)	9.33 (7.87)	7.94 (7.97)	7.00 (8.29)	25.0 [5.0, 44.9]	0.29 [0.03, 0.55]	–	–
Requested extension	19.21 (6.12)	12.55 (8.36)	10.11 (8.84)	8.47 (7.80)	32.5 [19.2, 45.9]	0.50 [0.23, 0.78]	–	–
Did not access booster	19.19 (6.55)	12.46 (8.60)	9.51 (8.39)	10.59 (9.28)	–	–	−11.4 [−34.8, 12.1]	−0.12 [−0.39, 0.14]
Accessed booster	17.72 (6.81)	9.66 (8.34)	7.88 (8.53)	6.61 (7.60)	–	–	16.1 [−2.4, 34.6]	0.16 [−0.10, 0.42]

PDSS-SR								
Did not request extension	8.23 (5.93)	4.35 (4.51)	3.04 (3.87)	3.01 (3.61)	30.8 [11.8, 49.9]	0.33 [0.06, 0.59]	–	–
Requested extension	9.35 (6.51)	5.75 (5.14)	4.54 (5.48)	4.04 (4.65)	29.8 [13.1, 46.4]	0.35 [0.07, 0.62]	–	–
Did not access booster	9.51 (6.69)	5.43 (4.88)	3.60 (4.43)	3.45 (4.02)	–	–	4.1 [−24.3, 32.5]	0.03 [−0.23, 0.30]
Accessed booster	7.09 (5.57)	4.01 (4.33)	3.09 (3.79)	2.98 (3.70)	–	–	3.5 [−19.7, 26.7]	0.03 [−0.23, 0.29]

SIAS-6/SPS-6								
Did not request extension	25.75 (10.63)	12.58 (10.64)	14.02 (12.14)	12.35 (11.40)	1.9 [−17.9, 21.7]	0.02 [−0.24, 0.28]	–	–
Requested extension	27.61 (9.47)	13.98 (10.05)	17.52 (12.96)	15.39 (11.78)	−10.1 [−27.3, 7.2]	−0.13 [−0.40, 0.15]	–	–
Did not access booster	27.76 (10.46)	14.81 (10.99)	16.54 (12.82)	16.37 (13.09)	–	–	1.1 [−17.6, 19.7]	0.01 [−0.25, 0.28]
Accessed booster	25.04 (9.92)	10.73 (9.40)	13.53 (12.25)	11.51 (11.04)	–	–	14.9 [−0.3, 30.2]	0.17 [−0.09, 0.43]

PCL-5								
Did not request extension	32.45 (19.73)	21.64 (16.83)	13.98 (14.64)	12.76 (12.97)	41.0 [24.0, 58.1]	0.59 [0.23, 0.95]	–	–
Requested extension	32.16 (15.84)	26.46 (17.76)	21.44 (19.31)	16.95 (16.62)	35.9 [21.0, 50.9]	0.55 [0.22, 0.88]	–	–
Did not access booster	36.70 (18.85)	29.62 (17.68)	17.97 (17.86)	15.70 (14.79)	–	–	12.6 [−11.1, 36.4]	0.14 [−0.19, 0.47]
Accessed booster	32.41 (15.61)	21.89 (15.81)	18.50 (18.38)	15.80 (15.59)	–	–	14.6 [−5.8, 35.0]	0.16 [−0.18, 0.49]

EQ VAS								
Did not request extension	58.75 (18.78)	72.75 (21.01)	72.73 (16.11)	69.16 (19.35)	−13.2 [−29.5, 3.2]	−0.18 [−0.44, 0.08]	–	–
Requested extension	54.66 (20.53)	70.10 (17.50)	66.77 (16.84)	69.50 (17.07)	−2.0 [−13.9, 9.9]	−0.03 [−0.31, 0.24]	–	–
Did not access booster	56.88 (21.02)	65.61 (23.20)	64.86 (17.74)	63.54 (19.54)	–	–	−3.8 [−18.8, 11.3]	−0.07 [−0.34, 0.19]
Accessed booster	59.19 (19.45)	74.66 (19.04)	73.94 (14.74)	73.79 (14.92)	–	–	−0.6 [−11.6, 10.5]	−0.01 [−0.27, 0.25]

Note. PHQ-9 = Patient Health Questionnaire-9; GAD-7 = Generalized Anxiety Disorder-7; SDS = Sheehan Disability Scale; PDSS-SR = Panic Disorder Severity Scale Self- Report; SIAS-6 = Social Interaction Anxiety Scale 6-item; SPS-6 = Social Phobia Scale 6-item; PCL-5 = PTSD Checklist for DSM-5; EQ VAS = EuroQol Visual Analogue Scale. Comparisons of “Did not request extension” and “Requested extension” included all patients in ICBT-extension and ICBT-extension + booster. Comparisons of “Did not access booster” and “Accessed booster” included all patients in ICBT-booster and ICBT-extension + booster.

### 3.5 Secondary outcomes

Table 2 also summarizes the means, standard deviations, percentage reductions, and Cohen's *d* effect sizes for the secondary measures (i.e., SDS, PDSS-SR, SIAS-6/SPS-6, PCL-5, and EQ-VAS) shown by factor. Supplementary Table 1 shows the same information by each treatment condition. As with the primary measures, the GEE analysis revealed statistically significant time effects for all variables (*p* < .001). From pre-treatment to week 26, the effect sizes were large for all treatment conditions on the SDS (Cohen's *d*: 1.27–1.44), PDSS-SR (Cohen's *d*: 0.97–1.03), SIAS-6/SPS-6 (Cohen's *d*: 1.10–1.22), PCL-5 (Cohen's *d*: 1.04–1.25), and medium for the EQ-VAS (Cohen's *d*: 0.56–0.69). There was also a significant time ∗ extension ∗ booster interaction at week-26 for the EQ-VAS (*p* = .08), but not other secondary measures (*p* > .12). No time ∗ extension or time ∗ booster interactions were found at week 26 on any of the secondary measures (*p* > .15). Pairwise comparisons involving the 3-way interaction on the EQ-VAS showed the EQ-VAS improvements at week 26 were larger in the ICBT-extension condition (35.3%) than ICBT (23.1%, *p* = .08) or ICBT extension + booster (22.9%, *p* = .07). Other differences were not significant.

Table 3 includes comparisons on secondary measures between patients who did and did not use the extension or booster. There were no significant differences in proportional changes from week 8 to 26 for patients who requested the extension (*p* range: 0.27–0.93) compared to patients who did not request the extension. Patients who accessed the booster had larger improvements from week 16 to 26 on the SDS (16.1%) than patients who did not access the booster (−11.4%, *p* = .06). There were no other significant differences found (*p* range: 0.16–0.88).

For the interested reader, Supplementary Table 2 shows reliable change on primary outcomes at 26 weeks. Across conditions, 52.1% of patients recovered on the PHQ-9, 56.0% recovered on the GAD-7, 2.1% of patients deteriorated on the PHQ-9, and 2.4% deteriorated on the GAD-7 at week 26. There were no differences observed among the conditions (*p* range: 0.25–0.98). Supplementary Table 3 shows patient medication and health service use at pre-treatment and at 26 weeks. The only difference that was observed was visits to family physician/nurse/other health professionals reduced from 70.3% to 35.0% (*p* < .001).

### 3.6 Treatment engagement

Treatment engagement data at 26 weeks is included in Table 4. Overall, 82.9% (*n* = 360) of patients completed at least 4 lessons, and 71.2% (*n* = 309) completed all 5 lessons. The majority of patients completed primary outcome measures at post-treatment (79.3%, *n* = 344), 12-week (67.7%, *n* = 294), and 26-week (67.5%, *n* = 293) follow-up. No group differences were found.

**Table 4:** Treatment engagement by treatment condition.

Variable	All participants (*N* = 434)		ICBT (*n* = 108)		ICBT-extension (*n* = 103)		ICBT-booster (*n* = 110)		ICBT-extension + booster (*n* = 113)	
	*n*	%	*n*	%	*n*	%	*n*	%	*n*	%
Completion of 4 lessons	360	82.9	86	79.6	93	90.3	88	80.0	93	82.3
Completion of 5 lessons	309	71.2	72	66.7	77	74.8	79	71.8	81	71.7
Completion of post-treatment primary measures	344	79.3	79	73.1	87	84.5	87	79.1	91	80.5
Completion of 16-week primary measures	294	67.7	80	74.1	72	69.9	67	60.9	75	66.4
Completion of 26-week primary measures	293	67.5	68	63.0	71	68.9	72	65.5	82	72.6
Mean days between first and last log-in (*SD*)	77.74 (46.93)	–	63.19 (44.30)	–	67.69 (35.08)	–	91.39 (52.93)	–	86.85 (47.55)	–
Mean number of log-ins (*SD*)	22.41 (19.76)	–	21.75 (19.99)	–	20.41 (12.95)	–	23.57 (19.42)	–	25.65 (23.63)	–
Mean messages from therapist to patient (*SD*)	10.71 (2.66)	–	9.15 (1.80)	–	10.94 (2.51)	–	10.56 (1.97)	–	12.14 (3.19)	–
Mean messages from patient to therapist (SD)	4.22 (3.85)	–	3.80 (2.98)	–	4.56 (4.43)	–	3.96 (3.87)	–	4.55 (4.00)	–
Mean phone calls with therapist (*SD*)	1.50 (1.73)	–	1.31 (1.52)	–	1.60 (1.95)	–	1.44 (1.54)	–	1.66 (1.88)	–
Mean total minutes therapists spent per client	124 (62)	–	109 (52)	–	126 (61)	–	121 (58)	–	140 (71)	–

Note. ICBT = Internet-delivered Cognitive Behaviour Therapy.

A main effect was found for extension (extension *M* = 11.60, *SD* = 2.97; no extension *M* = 9.91, *SD* = 2.07; *F*_(1,430)_ = 49.29 *p* < .001) and for booster (booster *M* = 4.46, *SD* = 4.06; no booster *M* = 4.23, *SD* = 3.65; *F*_(1,430)_ = 37.63 *p* < .001), with patients assigned to either condition receiving more messages from therapists than those who were not. A main effect was also found for extension on number of messages patients sent to their therapists, *F*_(1,430)_ = 4.61 *p* = .03, with extension patients sending more messages than those who were not assigned to extension (extension *M* = 4.75, *SD* = 4.23; no extension *M* = 3.95, *SD* = 3.43). Further, those who were assigned to booster had a larger number of days between their first and last login (booster *M* = 89.09, *SD* = 50.21; no booster *M* = 65.38, *SD* = 40.03; *F*_(1,430)_ = 29.23 *p* < .001). No main effects were found for the number of logins (*p* range: 0.15–0.85) or for the number of phone calls from therapists to patients (*p* range: 0.12–0.56). No significant interaction effects were found for any of the treatment engagement variables (*p* range: 0.30–0.89).

### 3.7 Treatment experiences by those using and not using extension and booster

Patients' treatment experiences rated at 26-week follow-up are summarized in Table 5. Most patients were either ‘Satisfied’ or ‘Very Satisfied’ with the course overall (85.3%, *n* = 243), with the website (90.5%. *n* = 258), with materials (88.1%. *n* = 251), with emails (86.7%. *n* = 247) and telephone calls if applicable (78.3%, *n* = 137), and either agreed or strongly agreed that their therapist was knowledgeable and competent (81.8%, *n* = 300). Most patients indicated increased confidence in their ability to manage their symptoms (81.8%, *n* = 233), as well as increased motivation to seek treatment if needed in the future (77.9%, *n* = 222). Nearly all patients indicated that the course was worth their time (93.7%, *n* = 267) and that they would recommend it to a friend (93.7%, *n* = 267). There were no main effects or interactions found related to patients' treatment experiences at 26-week follow-up (*p* range: 0.10–0.91). These indicators were similar at post-treatment and 16-weeks and thus are not reported in Table 5.

**Table 5:** Treatment perceptions/experiences by participants completing 26-week follow-up measures by treatment condition.

Variable	All participants (*n* = 340)		ICBT (*n* = 79)		ICBT-extension (*n* = 87)		ICBT-booster (*n* = 84)		ICBT-extension + booster (*n* = 90)	
	*n*	%	*n*	%	*n*	%	*n*	%	*n*	%
Week 26 ratings^1^	(*n* = 285)		(*n* = 67)		(*n* = 70)		(*n* = 69)		(*n* = 79)	
Mean credibility (*SD*)	22.42 (4.85)	–	21.91 (5.21)	–	22.61 (5.47)	–	23.14 (3.65)	–	22.05 (4.87)	–
Satisfied/very satisfied overall	243	85.3	58	86.6	57	81.4	62	89.9	66	83.5
Satisfied/very satisfied with the website	258	90.5	60	89.6	64	91.4	63	91.3	71	89.9
Satisfied/very satisfied with materials	251	88.1	60	89.6	60	85.7	63	91.3	68	60.2
Agreed/strongly agreed therapist knowledgeable and competent	233	81.8	55	82.1	58	82.9	51	73.9	69	87.3
Satisfied/very satisfied with emails	247	86.7	58	86.6	60	85.7	60	87.0	69	87.3
Satisfied/very satisfied with telephone calls^2^	137	78.3	34	82.9	29	70.7	37	78.7	37	80.4
Increased/greatly increased confidence	233	81.8	53	79.1	57	81.4	59	85.5	64	81.0
Increased/greatly increased motivation for other treatment	222	77.9	50	74.6	57	81.4	55	79.7	60	75.9
Course was worth time (%)	267	93.7	61	91.0	65	92.9	66	95.7	75	94.9
Would recommend course to friend (%)	267	93.7	62	92.5	66	94.3	68	98.6	71	89.9
Negative effects										
Reported negative effects	24	8.4	5	7.5	9	12.9	3	4.3	7	8.9
Negative effect impact (0–3)	2.08 (1.18)	–	2.00 (0.71)	–	2.00 (1.32)	–	2.00 (1.73)	–	2.29 (1.25)	–
Negative effect continues to have negative impact (0–3)	1.42 (1.06)	–	1.20 (0.45)	–	1.33 (1.32)	–	1.67 (0.58)	–	1.57 (1.27)	–

Note. ICBT = Internet-delivered Cognitive Behaviour Therapy.

1 Treatment ratings were also compared at Week 8 and Week 16, with no significant differences found over time or between groups (*p*: 0.11–0.99).

2 Percentages and statistical significance exclude patients who indicated ‘Not applicable’.

Very few patients reported experiencing an unwanted negative effect at post-treatment (10.4%; *n* = 35/336), 16-week (9.8%; *n* = 28/285), or 26-week (8.4%, *n* = 24/285) follow-up. At 26-week follow-up, the mean impact of the negative effect was 2.08 (*SD* = 1.18) and the mean rating of how much the negative effect continued to impact patients was 1.42 (*SD* = 1.42). The most common negative effect when considering all time periods was an increase in existing symptoms (n = 30/56, 53.6%), followed by negative thoughts about time lost or participation in the course (*n* = 9/56, 16.1%), new negative emotions (*n* = 7/56, 12.5%), and no specific reason provided (*n* = 10/56, 17.9%). No group differences or interactions were found.

Sub-analyses were conducted to compare treatment experiences at 26-week follow-up between patients who requested the extension and those who did not, and patients who accessed booster compared to those who did not. No significant differences were found in patients' treatment experiences between those who requested extension compared to those who did not at 26-week follow-up (*p* range: 0.19–0.95). On the other hand, at 26-week follow-up, patients who completed the booster reported greater satisfaction with the treatment overall (92.8% vs 74.5%; ꭙ^2^ (1, *N* = 148) = 9.55, *p* = .002), with the treatment website (96.9% vs 78.4%; ꭙ^2^ (1, *N* = 148) = 13.32, *p* < .001), and with the treatment materials (94.8% vs 76.5%; ꭙ^2^ (1, *N* = 148) = 11.10, *p* < .001), compared to those who did not complete the booster. Additionally, at 26-week follow-up, patients who accessed the booster reported the treatment was more credible (*M* = 23.20; *SD* = 3.68 vs *M* = 21.35; *SD* = 5.28); *F*_(1,146)_ = 6.16, *p* = .01), had increased confidence in their ability to manage their symptoms (88.7% vs 72.5%; ꭙ^2^ (1, N = 148) = 6.18, *p* = .01), and were more likely to indicate that the course was worth their time (97.9% vs 90.2%; ꭙ^2^ (1, N = 148) = 4.45, *p* = .04), and that they would recommend the course to a friend (97.9% vs 86.3%; ꭙ^2^ (1, N = 148) = 7.96, *p* = .005) compared to those who did not access the booster.

We conducted additional analyses to examine if at post-treatment, patients who requested the extension compared to those who did not were more likely to have scores in the clinical range on the PHQ-9 or the GAD-7 at post-treatment and found that more clients who requested the extension were in the clinical range on either measure compared to those who did not request the extension (37.2% vs 20.2%; ꭙ^2^ (1, *N* = 178) = 6.20, *p* = .01). In contrast, fewer clients who accessed the booster were in the clinical range on the PHQ-9 or GAD-7 at post-treatment compared to those who did not access the booster (21.1% vs 33.8%; ꭙ^2^ (1, *N* = 179) = 3.55, *p* = .06). Mean homework reflection ratings for each lesson are summarized in Table 6 comparing ratings for those who requested extension or not and those who used booster or not. In terms of extension, patients who requested the extension compared to those who did not put less effort into Lessons 2, 3 and 5. Patients who requested the extension also rated the skills from Lessons 1, 4 and 5 as more difficult than those who did not request the extension and also rated the skills from Lesson 1 as more helpful than patients who did not request the extension. In terms of use of the booster, across all five lessons, patients who accessed the booster rated putting more effort into practicing the lesson's skills than those who did not complete the booster. Finally, patients who accessed the booster rated the helpfulness of the skills in Lesson 3 and 5 as higher than those who did not complete the booster.

**Table 6:** Homework reflection ratings by those who requested or did not request extension and those who used or did not use booster.

Variable	All participants (*N* = 434)	Extension (*n* = 216)		Statistical significance	Booster (*n* = 223)		Statistical significance
		Not requested (*n* = 113)	Requested (*n* = 103)		Not used (*n* = 109)	Used (*n* = 114)	
Lesson 1 ratings^a^	(*n* = 379)	(*n* = 91)	(*n* = 99)		(*n* = 86)	(*n* = 108)	
Effort put into the skills (SD)	1.86 (0.86)	1.92 (0.75)	1.75 (0.89)	*F*_(1,188)_ = 2.16, *p* = .14	1.73 (0.89)	2.05 (0.79)	*F*_(1,192)_ = 6.88, *p* < .01
Difficulty practicing skills (SD)	0.72 (0.86)	0.49 (0.66)	0.93 (1.05)	*F*_(1,135)_ = 8.73, *p* < .01	0.69 (0.89)	0.71 (0.87)	*F*_(1,143)_ = 0.04, *p* = .85
Understand lesson (SD)	3.11 (0.65)	3.16 (0.67)	3.07 (0.62)	*F*_(1,161)_ = 0.80, *p* = .37	3.13 (0.66)	3.13 (0.58)	*F*_(1,166)_ = 0.00, *p* = .99
Helpfulness of skill (SD)	2.07 (0.88)	1.95 (0.93)	2.29 (0.84)	*F*_(1,128)_ = 4.75, *p* = .03	1.98 (0.84)	2.14 (0.95)	*F*_(1,134)_ = 1.04, *p* = .31
Lesson 2 ratings	(*n* = 338)	(*n* = 81)	(*n* = 85)		(*n* = 70)	(*n* = 110)	
Effort put into the skills (SD)	1.76 (0.87)	1.88 (0.80)	1.65 (0.87)	*F*_(1,164)_ = 3.14, *p* = .08	1.61 (1.04)	1.94 (0.81)	*F*_(1,178)_ = 5.44, *p* = .02
Difficulty practicing skills (SD)	1.23 (0.94)	1.26 (0.98)	1.37 (0.99)	*F*_(1,142)_ = 0.49, *p* = .49	1.28 (1.01)	1.21 (0.92)	*F*_(1,153)_ = 0.23, *p* = .63
Understand lesson (SD)	2.84 (0.72)	2.95 (0.58)	2.75 (0.86)	*F*_(1,138)_ = 2.63, *p* = .11	2.85 (0.69)	2.80 (0.79)	*F*_(1,147)_ = 0.13, *p* = .72
Helpfulness of skill (SD)	2.20 (0.91)	2.33 (0.86)	2.13 (0.89)	*F*_(1,128)_ = 2.63, *p* = .11	2.10 (0.96)	2.31 (0.87)	*F*_(1,140)_ = 1.73, *p* = .19
Lesson 3 ratings	(*n* = 252)	(*n* = 49)	(*n* = 81)		(*n* = 42)	(*n* = 80)	
Effort put into the skills (SD)	1.49 (0.88)	1.61 (0.76)	1.32 (0.79)	*F*_(1,128)_ = 4.29, *p* = .04	1.17 (0.94)	1.65 (0.78)	*F*_(1,120)_ = 9.19, *p* < .01
Difficulty practicing skills (SD)	1.30 (1.05)	1.05 (0.89)	1.46 (0.99)	*F*_(1,87)_ = 4.07, *p* = .05	1.26 (1.14)	1.31 (1.13)	*F*_(1,82)_ = 0.03, *p* = .86
Understand lesson (SD)	2.83 (0.73)	2.78 (0.73)	2.77 (0.61)	*F*_(1,92)_ = 0.00, *p* = .96	2.67 (0.76)	2.88 (0.60)	*F*_(1,88)_ = 1.92, *p* = .17
Helpfulness of skill (SD)	2.16 (0.97)	2.19 (0.97)	2.10 (0.83)	*F*_(1,83)_ = 0.19, *p* = .66	1.86 (0.94)	2.29 (0.92)	*F*_(1,78)_ = 3.45, *p* = .07
Lesson 4 ratings	(*n* = 286)	(*n* = 63)	(*n* = 83)		(*n* = 40)	(*n* = 99)	
Effort put into the skills (SD)	1.48 (0.85)	1.52 (0.72)	1.31 (0.84)	*F*_(1,144)_ = 2.55, *p* = .11	1.15 (0.77)	1.62 (0.90)	*F*_(1,137)_ = 8.27, *p* < .01
Difficulty practicing skills (SD)	1.19 (1.01)	0.94 (0.79)	1.46 (1.12)	*F*_(1,114)_ = 7.94, *p* < .01	1.14 (1.04)	1.29 (1.11)	*F*_(1,108)_ = 0.39, *p* = .53
Understand lesson (SD)	2.78 (0.81)	2.88 (0.80)	2.73 (0.81)	*F*_(1,107)_ = 0.96, *p* = .33	2.74 (1.01)	2.76 (0.76)	*F*_(1,104)_ = 0.01, *p* = .92
Helpfulness of skill (SD)	1.98 (0.98)	2.00 (0.91)	1.90 (0.89)	*F*_(1,95)_ = 0.30, *p* = .59	2.00 (1.05)	2.00 (0.92)	*F*_(1,94)_ = 0.00, *p* = 1.00
Lesson 5 ratings	(*n* = 242)	(*n* = 57)	(*n* = 60)		(*n* = 37)	(*n* = 91)	
Effort put into the skills (SD)	1.53 (0.87)	1.60 (0.68)	1.35 (0.88)	*F*_(1,115)_ = 2.86, *p* = .09	1.16 (0.80)	1.69 (0.85)	*F*_(1,126)_ = 10.54, *p* < .01
Difficulty practicing skills (SD)	0.90 (0.97)	0.57 (0.73)	1.33 (1.11)	*F*_(1,75)_ = 13.33, *p* < .01	0.68 (0.84)	1.04 (1.03)	*F*_(1,90)_ = 2.24, *p* = .14
Understand lesson (SD)	3.05 (0.68)	3.10 (0.63)	2.97 (0.64)	*F*_(1,84)_ = 0.91, *p* = .34	2.82 (0.73)	3.03 (0.67)	*F*_(1,93)_ = 1.59, *p* = .21
Helpfulness of skill (SD)	2.37 (0.97)	2.30 (0.94)	2.29 (1.05)	*F*_(1,69)_ = 0.01, *p* = .95	2.00 (0.95)	2.50 (0.94)	*F*_(1,81)_ = 4.44, *p* = .04

a Effort ratings were based on a scale ranging from 0 (*None*) to 4 (*A great deal*). Difficulty practicing skills, understanding of the lesson, and helpfulness of the skill were rated on a scale from 0 (*Not at all*) to 4 (*Extremely*).

### 3.8 Therapist experiences

#### 3.8.1 Timing

The total amount of time that therapists spent across treatment is reported in Table 4. A two-way ANOVA yielded a main effect for extension, *F*_(1,433)_ = 10.20, *p* = .002, whereby therapists spent more time on patients randomized to extension (*M*: 133, *SD*: 66 min) than those who were not (*M*: 115, *SD*: 55 min). A main effect was also found for randomization to booster, *F*_(1,433)_ = 4.91, *p* = .03, whereby therapists spent more time on patients randomized to booster (*M*: 130, *SD*: 66 min) than on patients who were not (*M*: 117, *SD*: 57 min). The interaction effect was not significant (*p* = .89).

#### 3.8.2 Observations

Both benefits and challenges of each condition were noted by therapists. Benefits to those who received extension included a perceived facilitation of understanding for actively engaged patients, an opportunity for patients to catch up if they had fallen behind during the 8-week ICBT course, and to manage any setbacks as needed. Challenges with the extension were experienced by therapists when patients reported unclear goals, and patients were not consistently engaged (e.g., not logging in). Therapists highlighted that additional time was needed to compose messages to patients compared to earlier weeks in treatment for various reasons (i.e., messages were highly individualized in terms of content patients were working on, providing instructions about the nature of the extension, and patients straying from the course materials due to no new content during the extension). Further, several logistical concerns were identified, such as patients missing the opportunity to choose an extension (i.e., if they did not login during the two weeks that the option was available to them), therapists' inability to discuss the extension prior to week 6, and a lack of automated email reminders for patients during the extension.

In terms of the booster, therapists perceived this condition as helpful for patients who required additional support or follow-up after the standard 8-week treatment. Therapists commented on how patients found it helpful to review skills during the booster. Therapists appreciated that the booster did not require them to call patients who were not actively engaged with the booster. Some of the challenges associated with the booster were that therapists had to write an individualized email to all patients in this condition in the event they logged in. This required them to review the patient case notes, which took extra time as it had been between 4 and 8 weeks since their last contact.

In terms of ICBT-extension + booster condition, therapists noted that the combination had the same benefits as noted above related to the extension and the booster. Unique challenges that therapists identified in the combined condition were that the booster was only four weeks after the extension, instructions about the combined condition were cumbersome, and therapists had to increase their amount of contact with patients from eight contacts to as many as 15 contacts without any clear benefits to patients.

## 4 Discussion

In this study, we contribute to pragmatic knowledge of ICBT in routine practice by examining how many and which patients make use of an extension, a booster or the combination of the two as well as whether there are benefits associated with offering these treatment options. In routine practice, patients have requested having greater flexibility when it comes to the end of treatment, specifically in the form of an extension of therapist support (Hadjistavropoulos et al., 2018a). Further, findings from face-to-face CBT suggest that booster sessions offered at some point post-treatment can maintain treatment improvements (Whisman, 1990). Based on existing literature, we predicted that an extension and a booster (and the combination) would result in greater improvements on depression and anxiety as well as greater engagement and patient satisfaction at 26-week follow-up.

### 4.1 Patient use of extension and booster

Overall, about half the patients made use of an extension and about half made use of a booster. Extension participation, however, was lower when patients were offered both an extension and a booster. In this case, only 26.5% of patients opted to use both the extension and the booster. There were some variables that differentiated those who made use of the extension compared to those who did not (i.e., older age, taking psychotropic medication, concurrent use of mental health services, more severe pre-treatment mental health-related disability, clinically significant symptoms at post-treatment). Similarly, there were differences between those who did and do not use the booster (i.e., older age, less severe pre-treatment symptoms of depression, anxiety, panic, and social phobia, fewer pre-treatment measures within the clinically significant range, lower post-treatment clinically significant depression and or anxiety). Of interest, factors that predicted requests for extension were similar to factors that have been found in the literature to predict ICBT non-completion, and factors that predicted use of a booster were similar to factors that have been found to predict ICBT treatment completion. Specifically, past researchers have found that higher severity of pre-treatment symptoms/comorbid depression (Mathiasen et al., 2018), psychotropic medications (El Alaoui et al., 2016; Hadjistavropoulos et al., 2016b), and use of psychiatric care (Hadjistavropoulos et al., 2016b) predict non-completion of ICBT, which were similar predictors of requests for the extension in this study. Conversely, older age and lower psychological distress have been found to predict treatment completion (Edmonds et al., 2018) and were similar to predictors of use of the booster in this study. Of note, unlike the current study, previous studies on boosters after face-to-face therapy have failed to identify any pre-treatment predictors of booster usage (e.g., following face-to-face CBT for panic disorder, see Craske et al., 2006).

It also appeared that experiences with the five lessons were related to use of the extension and the booster lesson. Specifically, patients who requested the extension indicated putting less effort into some lessons. For these patients, the extension appeared to provide them with the opportunity to “catch up” on content if they were not able to complete all five lessons within the standard 8-week treatment period. These relationships are particularly noteworthy as with future research it may be possible for therapists to use patient ratings (i.e., level of effort or difficulty of skills) to identify patients who would benefit from additional support prior to end of treatment and the patient requesting extension. A different picture was apparent among patients who used the booster. In this case, those who opted to use the booster reported putting more effort into all five lessons; rated the helpfulness of two of the lessons higher; reported greater satisfaction with the treatment overall, with the treatment platform, and with the treatment materials; were more likely to report that the treatment was worth their time and that they would recommend it to a friend; had greater confidence in their ability to manage their symptoms; and rated the treatment as more credible overall than patients who did not use the booster. For patients who utilized the booster, it seemed that their treatment experiences played an important role in their desire to make use of the booster additional support offered. This finding is consistent with previous studies on booster sessions (i.e., Baker and Wilson, 1985) that found that the success of the initial intervention is an important predictor of booster outcomes.

### 4.2 Patient outcomes

While use of the extension and the booster were quite common, the current study did not identify major differences in outcomes across groups. Large improvements were seen on measures of depression (within Cohen's *d* = 1.36–1.46; avg. % reduction ≥ 53.2%), generalized anxiety (within Cohen's *d* = 1.53–1.69; avg. % reduction ≥ 57.1%), disability (within Cohen's *d* = 1.27–1.44; avg. % reduction ≥ 53.6%), panic (within Cohen's *d* = 0.99–1.03; avg. % reduction ≥ 60.0%), social anxiety/phobia (within Cohen's *d* = 1.10–1.14; avg. % reduction ≥ 47.3%), and posttraumatic stress (within Cohen's *d* = 1.04–1.25; avg. % reduction ≥ 53.4%) at 26-week follow-up. Moderate effects were found for improvements in patients' ratings of their overall health on the EQ-VAS (within Cohen's *d* = 0.56–0.69; avg. % improvement ≥ 25.5%). In general, the findings from this trial were consistent with previous trials of transdiagnostic ICBT (e.g., Dear et al., 2015; Hadjistavropoulos et al., 2016, Hadjistavropoulos et al., 2017, Hadjistavropoulos et al., 2020, Hadjistavropoulos et al., 2020; Titov et al., 2015). Effect sizes were large from pre-treatment to 26-week follow-up on all primary and secondary measures (with the exception of the EQ-VAS).

Given participation levels in the extension and the booster, lack of differences in these conditions is perhaps not surprising, and may be related to insufficient power to detect differences. It may be that effects in patients who used the extension or the booster were diluted in the group averages by either patients who did not use the extension or the booster or by patients who had low pre-treatment symptoms. In the end, the design proved to be complex with ICBT-extension being made up of those who did or did not use the extension, and similarly, ICBT-booster being made up of those who did or did not use the booster. Further, even among those who chose the extension or accessed the booster, a significant subgroup did not engage with their therapists during the extension (35.0%) or the booster (33.0%). A stronger test of the effects of extension and booster in the future would be to identify patients who want or need the extension (e.g., continue to have elevated symptoms post-treatment) and then to randomly assign patients to extension or not, and, similarly, to identify patients who want or need (e.g., continue to have elevated symptoms post-treatment) the booster and randomly assign patients to booster or not. In hindsight, knowing now how many actually use the extension or the booster, the alternate design would have allowed for better evaluation of the benefits of the extension and the booster.

Overall, while no major differences were found, there were some trends in the data to suggest that further research may be worthwhile. There was some evidence that patients who received ICBT extension + booster had worse outcomes than those who received ICBT-booster on the GAD-7. Furthermore, on the EQ-VAS those in ICBT-extension + booster and those in the ICBT only reported lower improvements than those in the ICBT-extension. These trends could suggest that ultimately offering the extension and the booster in the same protocol is less preferable than the booster in the case of GAD-7 and less preferable than the extension in the case of the EQ-VAS. Given that the effects were not strong or consistent across measures we highlight them here only to suggest that further research may be warranted and to provide some caution against offering both extension and booster.

Overall, at this time, we cannot conclude that the extension of the booster improve outcomes, but the findings related to how many patients want and who wants the extension and the booster set the stage for future research on this topic. It is compelling that ~50% of patients wanted the extension and that this was particularly prominent for those with higher pre-treatment and post-treatment symptom severity suggesting that there is a need to identify what would ultimately help these patients at the end of treatment who still want assistance.

### 4.3 Therapist experiences

From an implementation perspective, we were interested in how the extension and the booster conditions would impact therapists since therapist experiences have potential to influence implementation efforts in routine care (Hadjistavropoulos et al., 2017a). Therapists spent an average of 18 min (14.5%) more on patients offered the extension and 13 min (10.5%) more on patients offered the booster compared to patients who were not randomized to these conditions, respectively. In the case of ICBT-extension + booster, about one-quarter made use of both forms of additional support. Understanding amount of time required provides useful information to those who deliver ICBT in the event they want to offer extensions or boosters to meet consumer demand, although it is not yet known whether these services add benefit. Overall, the amount of time required to offer the service is quite minimal. Therapist observations suggested a range of benefits and drawbacks to both the extension and the booster. Therapists perceived the conditions to be beneficial in that they increased the opportunity to assist patients who wanted extra services. For some of the patients, therapists noted that the extension and the booster appeared to benefit patient understanding and skill development and this was rewarding for therapists. On the negative side, therapists noted more challenges with how the extension was implemented compared to the booster. In the case of the extension, therapists expressed finding it difficult to keep emailing patients who ultimately did not use the extension even though they requested it. In the future, if extensions are offered, therapists would recommend offering support only if patients specifically email during an extension period. They also reported finding it more challenging to email patients when there was no new treatment content; therapists suggested it would be preferable to have patients set clearer goals for the extension period. In the case of the booster, therapists indicated the structure of the booster was easier to implement given that there was new content available and also there was a two-week versus four-week period of providing support. Therapists also preferred that they did not have to reach out to patients who did not reach out to them. The greatest challenge with the booster was that therapists were required to send a brief personalized email to patients that would be waiting for patients in the event that they completed the booster (e.g., an email asking patients how they have been). Therapists suggested automating this initial message. Given that about half of patients did not ultimately read this personalized message, therapists rightly perceived this effort to be pointless for many patients.

### 4.4 Limitations and future directions

There are notable limitations to this trial which provide directions for future research. The study results may not generalize to other routine care clinics and further study of extensions and boosters in other settings is important. We were missing data from 21.7% of patients at post-treatment and 32.5% of patients at 26-week follow-up. It is possible that there are subgroups of patients who benefit from an extension and a booster lesson that our sample size did not allow us to identify, especially since some of individuals in this trial had low symptom levels at pre-treatment, which may have diluted effects. For example, it is possible that an extension and a booster may show greater benefit if all patients had scores in the clinical range at pre-treatment. It is also possible that an extension and booster could be useful for individuals who complete all activities, described as “strivers”, as has been described in past qualitative literature (Bendelin et al., 2011). It is also possible, however, that these patients are already benefiting from ICBT as it is currently delivered and the addition of an extension and a booster does not add to already positive outcomes. In terms of the extension and the booster, participation in these conditions could be a function of desirability effects (Sabourin et al., 1989), whereby patients who wish to be perceived positively by their therapists opt for participation without actually desiring these conditions. In the future, it may be valuable to randomly assign those who need extension and boosters based on post-treatment scores to receive it or not. The combined extension and booster condition resulted in a highly variable treatment condition (~27% received both conditions, 15% extension, 25% booster, and 34% ICBT) limiting conclusions that can be drawn about outcomes related to this condition. Longer term follow-up may have identified benefits of the extension and booster that were not apparent at 26-week follow-up. For example, within the Andersson et al. (2014) study on booster sessions for OCD, participants' general functioning and risk of relapse was significantly lower at 24 months compared to those who did not receive booster sessions. In terms of other limitations, it is possible that a more systematic approach to recording and coding therapist observations would have resulted in different observations (e.g., focus group). Finally, the treatment period for all patients in this trial coincided with the COVID-19 pandemic, and it is possible that patients experienced additional external stressors (e.g., loss of job, financial uncertainty, family or personal illness, death of a loved one; see World Health Organization, 2020) that may have impacted their ability to engage in the extension and the booster.

### 4.5 Strengths

In terms of strengths, this study represents a replication of past studies of transdiagnostic ICBT (e.g., Dear et al., 2015; Hadjistavropoulos et al., 2016, Hadjistavropoulos et al., 2017, Hadjistavropoulos et al., 2020, Hadjistavropoulos et al., 2020; Titov et al., 2015), and outcomes compare positively to other studies of ICBT for depression and anxiety (e.g., Andersson et al., 2019; Carlbring et al., 2018). The replication of strong outcomes, engagement and satisfaction is particularly valuable as the study took place during the COVID-19 pandemic. The study was conducted in routine care and the sample is comprised of patients who would normally seek and be offered ICBT. This study included 26-week follow-up and also assessed for diverse outcomes as well as patient engagement and various treatment experiences. The findings provide particularly useful information regarding how many and which patients want an extension and a booster as well as who actually makes use of these conditions and how much extra time is needed to offer support if implemented as described in this study. Furthermore, therapist suggestions for improving implementation of the extension and the booster provide directions for future implementation efforts.

## 5 Conclusions

The present study contributes to the existing literature on ICBT in routine care and fills gaps in research on use of extended support and booster lessons with ICBT. In both cases, interest in the extension and the booster was considerable, with about 50% of patients making use of the options. Older patients, in particular, were more likely to use the extension and the booster. Otherwise it appears different factors are associated with interest in an extension compared to the booster. In the case of the extension, there was greater interest among those who had greater severity (e.g., as suggested by several indicators such as taking medication, having concurrent mental health care, and more severe disability), and more challenges with the course (reported putting in less effort, finding skills more difficult). On the other hand, the booster was of greatest interest among those with lower symptom severity, and those who reported putting in more effort and having a positive experience with ICBT (as suggested by multiple indicators). While the study did not find that outcomes were improved when patients were offered the extension or the booster and also there were increased therapist costs associated with offering the extension and the booster, this represents important knowledge in routine practice as there is pressure for ICBT to continue to evolve and respond to consumer demands (e.g., Titov et al., 2019). It is possible that even though there are no clear benefits related to the extension or the booster in terms of outcomes and some added costs, this is still a service, especially with some minor adjustments taking into account therapist feedback that may be valuable to offer in routine care to address consumer demand. Nevertheless, it must be acknowledged at this time, that the added costs associated with the extension and the booster cannot yet be justified given that outcomes did not differ. This study provides an indication of the extent of the demand for these options and factors associated with the demand. The study also contributes to understanding therapist experiences with offering extensions and the booster most notably that it required ~15 min extra over ICBT alone to offer the extension or the booster. While therapists appreciated being able to provide this option to patients, they felt that extension was particularly challenging to implement when patients were unclear about what they wanted to gain from the extension and when patients requested but ultimately did not use the extension. The reality exists that 50% of patients had a desire for an extension and the question remains how to best address the needs of these patients. Other options to be explored in the future include focussing more on the nature of therapeutic support rather than on offering more support; for example using motivational interviewing earlier in treatment to assist with treatment engagement (Soucy et al., 2021). The booster also did not improve outcomes, but nevertheless was used by patients – perhaps offering this as self-directed manner would meet potential demand for this from consumers, but not increase costs. It would be worthwhile to continue to study booster lessons that are offered after different periods of time following the main course of treatment (e.g., one, three, or six-months post-treatment). Alternatively, therapists may assess the need for extension and boosters on a case-by-case basis, and offer it when symptoms remain elevated or it is perceived to be needed. This latter approach appears most consistent with a stepped-care model, whereby therapists step up the length of support or offer booster sessions as clinically indicated.

## Declaration of competing interest

The authors declare that they have no known competing financial interests or personal relationships that could have appeared to influence the work reported in this paper.

## Appendix A Supplementary data

Supplementary analyses based on treatment condition, reliable changes, and medication and health service use. Image 1

## Footnotes

1 Analyses showed no significant differences on baseline characteristics between patients assigned to the Online Therapy Unit and those assigned to the community mental health clinic.

2 For research planning purposes only, we administered the seven-item Insomnia Severity Index (ISI; Morin et al., 2011) at screening only.
