# Supplementary material for: A randomized factorial trial of internet-delivered cognitive behavioural therapy: An 8-week program with or without extended support and booster lesson
Source: Internet Interv. 2022 Feb 6;27:100499. doi: 10.1016/j.invent.2022.100499 (PMC8844810; doi:10.1016/j.invent.2022.100499)
Supplement: Supplementary file 1 — Supplementary analyses based on treatment condition, reliable changes, and medication and health service use. [file mmc1.docx]

| Supplementary Table 1. *Estimated marginal means, 95% confidence intervals, percentage changes, and effect sizes (Cohen’s d) for primary and secondary outcomes by treatment group using pooled imputations.* | | | | | | | | | | |
| --- | --- | --- | --- | --- | --- | --- | --- | --- | --- | --- |
|  | Estimated marginal means | | | |  | Percentage changes from pre-treatment | |  | Within-group effect sizes from pre-treatment | |
|  | pre-treatment | post-treatment | 16-week follow-up | 26-week follow up |  | to post-treatment | to 26-week follow-up |  | to post-treatment | to 26-week  follow-up |
| **Primary outcomes** | |  |  |  |  |  |  |  |  |  |
| **PHQ-9** |  |  |  |  |  |  |  |  |  |  |
| ICBT | 13.64 (5.49) | 7.93 (5.36) | 6.55 (4.72) | 6.67 (4.82) |  | 41.8  [33.2, 50.5] | 51.1  [42.8, 59.3] |  | 1.05  [0.76, 1.33] | 1.34  [1.05, 1.64] |
| ICBT-booster | 13.33 (5.47) | 5.84 (5.07) | 5.90 (4.82) | 5.21 (4.70) |  | 56.2  [47.9, 64.5] | 60.9  [53.1, 68.8] |  | 1.42  [1.12, 1.71] | 1.59  [1.28, 1.89] |
| ICBT-extension | 13.06 (5.23) | 6.52 (5.23) | 6.05 (4.96) | 5.80 (5.25) |  | 50.1  [41.8, 58.3] | 55.6  [46.8, 64.5] |  | 1.25  [0.95, 1.54] | 1.38  [1.08, 1.69] |
| ICBT-booster + extension | 14.51 (5.57) | 7.55 (6.24) | 6.67 (5.75) | 6.84 (5.65) |  | 48.0  [39.3, 56.7] | 52.9  [45.1, 60.7] |  | 1.17  [0.89, 1.46] | 1.36  [1.07, 1.65] |
| **GAD-7** |  |  |  |  |  |  |  |  |  |  |
| ICBT | 12.81 (5.01) | 7.56 (5.37) | 5.83 (4.51) | 5.70 (4.32) |  | 41.0  [31.8, 50.2] | 55.5  [47.5, 63.5] |  | 1.01  [0.72, 1.29] | 1.51  [1.21, 1.82] |
| ICBT-booster | 12.55 (4.66) | 5.56 (4.60) | 5.00 (4.14) | 4.50 (3.83) |  | 55.7  [47.8, 63.5] | 64.1  [56.7, 71.6] |  | 1.50  [1.20, 1.80] | 1.88  [1.56, 2.20] |
| ICBT-extension | 12.61 (5.19) | 6.33 (5.19) | 5.37 (4.90) | 5.04 (4.57) |  | 49.8  [41.4, 58.3] | 60.0  [52.1, 67.9] |  | 1.21  [0.91, 1.50] | 1.54  [1.23, 1.85] |
| ICBT-booster + extension | 13.27 (5.09) | 6.96 (5.80) | 6.22 (5.56) | 6.01 (4.92) |  | 47.6  [38.8, 56.3] | 54.7  [46.6, 62.8] |  | 1.15  [0.87, 1.43] | 1.44  [1.15, 1.74] |
| **Secondary Outcomes** | |  |  |  |  |  |  |  |  |  |
| **SDS** |  |  |  |  |  |  |  |  |  |  |
| ICBT | 17.78 (7.34) | 13.86 (7.86) | 9.28 (8.21) | 7.90 (8.11) |  | 22.1  [11.9, 32.2] | 55.6  [45.2, 65.9] |  | 0.51  [0.24, 0.78] | 1.27  [0.98, 1.57] |
| ICBT-booster | 18.15 (7.17) | 10.66 (8.70) | 8.40 (8.27) | 8.04 (8.67) |  | 41.2  [31.4, 51.1] | 55.7  [45.5, 65.9] |  | 0.94  [0.66, 1.21] | 1.27  [0.98, 1.56] |
| ICBT-extension | 17.83 (7.03) | 10.29 (8.01) | 9.02 (8.17) | 6.22 (7.07) |  | 42.3  [33.0, 51.5] | 65.1  [56.1, 74.2] |  | 1.00  [0.71, 1.29] | 1.64  [1.32, 1.96] |
| ICBT-booster + extension | 18.73 (6.24) | 11.39 (8.46) | 8.94 (8.73) | 9.06 (8.71) |  | 39.2  [30.2, 48.1] | 51.6  [41.8, 61.4] |  | 0.98  [0.71, 1.26] | 1.27  [0.99, 1.56] |
| **PDSS-SR** |  |  |  |  |  |  |  |  |  |  |
| ICBT | 8.14 (6.27) | 4.25 (4.61) | 3.66 (4.70) | 2.90 (3.82) |  | 47.8  [34.9, 60.7] | 64.4  [54.0, 74.7] |  | 0.70  [0.43, 0.98] | 1.01  [0.72, 1.29] |
| ICBT-booster | 7.72 (6.18) | 4.27 (4.41) | 3.29 (3.98) | 2.61 (3.43) |  | 44.7  [31.7, 57.7] | 66.2  [56.2, 76.2] |  | 0.64  [0.37, 0.91] | 1.02  [0.74, 1.30] |
| ICBT-extension | 8.71 (6.17) | 4.89 (4.87) | 4.17 (5.23) | 3.17 (4.15) |  | 43.8  [32.1, 55.6] | 63.6  [53.3, 73.9] |  | 0.68  [0.40, 0.96] | 1.05  [0.76, 1.34] |
| ICBT-booster + extension | 8.81 (6.30) | 5.14 (4.86) | 3.39 (4.27) | 3.80 (4.17) |  | 41.7  [30.3, 53.1] | 56.9  [46.4, 67.4] |  | 0.65  [0.38, 0.92] | 0.94  [0.66, 1.21] |
| **SIAS-6/SPS-6** |  |  |  |  |  |  |  |  |  |  |
| ICBT | 25.45 (9.97) | 12.53 (10.36) | 15.60 (11.84) | 13.17 (11.14) |  | 50.8  [42.2, 59.4] | 48.3  [38.7, 57.9] |  | 1.27  [0.98, 1.56] | 1.16  [0.87, 1.45] |
| ICBT-booster | 25.90 (10.69) | 11.73 (10.48) | 14.24 (12.36) | 12.83 (12.60) |  | 54.7  [46.4, 63.1] | 50.5  [40.4, 60.5] |  | 1.33  [1.04, 1.63] | 1.12  [0.83, 1.40] |
| ICBT-extension | 26.43 (10.44) | 12.76 (10.52) | 15.62 (12.46) | 12.57 (11.21) |  | 51.7  [43.6, 59.8] | 52.4  [43.3, 61.6] |  | 1.30  [1.00, 1.60] | 1.27  [0.97, 1.57] |
| ICBT-booster + extension | 26.83 (9.84) | 13.70 (10.25) | 15.75 (12.84) | 14.92 (11.99) |  | 49.0  [41.2, 56.7] | 44.4  [35.1, 53.7] |  | 1.30  [1.02, 1.59] | 1.08  [0.80, 1.36] |
| **PCL-5** |  |  |  |  |  |  |  |  |  |  |
| ICBT | 36.16 (18.69) | 26.33 (17.12) | 18.29 (16.15) | 16.45 (14.95) |  | 27.2  [14.9, 39.5] | 54.5  [43.2, 65.8] |  | 0.55  [0.21, 0.89] | 1.16  [0.80, 1.52] |
| ICBT-booster | 33.23 (16.02) | 23.83 (16.35) | 16.91 (17.22) | 12.95 (13.65) |  | 28.3  [14.9, 41.6] | 61.0  [49.4, 72.7] |  | 0.58  [0.24, 0.92] | 1.36  [0.99, 1.73] |
| ICBT-extension | 28.48 (15.78) | 20.60 (16.35) | 16.44 (16.26) | 11.35 (13.23) |  | 27.7  [13.4, 41.9] | 60.2  [47.3, 73.0] |  | 0.49  [0.14, 0.83] | 1.17  [0.80, 1.54] |
| ICBT-booster + extension | 35.89 (18.66) | 27.72 (17.86) | 19.55 (18.90) | 18.51 (16.10) |  | 22.8  [10.1, 35.4] | 48.4  [37.1, 59.8] |  | 0.44  [0.11, 0.78] | 0.99  [0.64, 1.34] |
| **EQ-VAS** |  |  |  |  |  |  |  |  |  |  |
| ICBT | 60.13 (17.78) | 71.92 (18.93) | 67.03 (17.61) | 69.36 (16.47) |  | 29.6  [19.2, 39.9] | 23.1  [13.6, 32.7] |  | 0.64  [0.37, 0.91] | 0.54  [0.27, 0.81] |
| ICBT-booster | 58.25 (20.88) | 71.36 (21.34) | 71.58 (16.02) | 70.07 (17.33) |  | 31.4  [20.4, 42.4] | 28.3  [18.5, 38.1] |  | 0.62  [0.35, 0.89] | 0.61  [0.34, 0.88] |
| ICBT-extension | 55.61 (19.76) | 74.06 (15.94) | 72.52 (15.46) | 71.29 (17.61) |  | 41.6  [33.7, 49.5] | 35.3  [26.3, 44.3] |  | 1.02  [0.73, 1.31] | 0.83  [0.55, 1.12] |
| ICBT-booster + extension | 57.88 (19.66) | 69.13 (21.93) | 67.49 (17.46) | 67.52 (18.71) |  | 26.7  [15.9, 37.4] | 22.9  [13.1, 32.7] |  | 0.54  [0.27, 0.80] | 0.50  [0.24, 0.77] |
| *Note.* ICBT = Internet-delivered Cognitive Behaviour Therapy; PHQ-9 = Patient Health Questionnaire-9; GAD-7 = Generalized Anxiety Disorder-7; SDS = Sheehan Disability Scale; PDSS-SR = Panic Disorder Severity Scale Self- Report; SIAS = Social Interaction Anxiety Scale; SPS = Social Phobia Scale; PCL-5 = PTSD Checklist for DSM-5; EQ VAS = EuroQol Visual Analogue Scale. | | | | | | | | | | |

| Supplementary Table 2. *Reliable recovery, reliable improvement,*  *no change, and deterioration on the PHQ-9 and GAD-7 at 26-week*  *follow-up using imputed data.* | |
| --- | --- |
|  | All Groups  (%) |
| **26-week follow-up** |  |
| **PHQ-9** |  |
| Reliable recovery | 52.1 |
| Reliable improvement | 63.0 |
| Reliable deterioration | 2.1 |
| No change | 34.9 |
| **GAD-7** |  |
| Reliable recovery | 56.0 |
| Reliable improvement | 75.7 |
| Reliable deterioration | 2.4 |
| No change | 22.0 |
| *Note.* PHQ-9 = Patient Health Questionnaire-9; GAD-7 = Generalized Anxiety Disorder-7. For the PHQ-9, reliable recovery was defined as patients scoring > 9 at pre-treatment, < 10 at week 26, and having a 6-point decrease or greater; reliable improvement was defined as a 6-point or greater decrease; deterioration was defined as a 6-point or greater increase; and no change was defined as not changing at least 6 points in either direction. The same approach was used for the GAD-7 except the critical value was 4. | |

| Supplementary Table 3. *Patient medication and health service use over the past three months at pre-treatment and 26-week follow-up by treatment condition.* | | | |
| --- | --- | --- | --- |
| Variable | | All groups  (N = 434) | |
|  |  |  |  |
|  |  | *n* | % |
| Psychotropic medication use | |  | |
|  | Pre-treatment | 260 | 59.9 |
|  | 26-week follow-up (*n* = 280) | 153 | 54.6 |
| Family doctor/walk-in/nurse/other health professional | | | |
|  | Pre-treatment | 305 | 70.3 |
|  | 26-week follow-up | 98 | 35.0 |
| Psychiatrist | |  |  |
|  | Pre-treatment | 78 | 18.0 |
|  | 26-week follow-up | 47 | 16.8 |
| Psychologist/counsellor/social worker | |  | |
|  | Pre-treatment | 106 | 24.4 |
|  | 26-week follow-up | 72 | 25.7 |
| Mental health treatment program^a^ | |  | |
|  | Pre-treatment | 25 | 5.7 |
|  | 26-week follow-up | 8 | 2.9 |
| Emergency room/ambulance/crisis service | | | |
|  | Pre-treatment | 8 | 1.8 |
|  | 26-week follow-up | 6 | 2.1 |
| ^a^Treatment programs include psychiatric day or part-time treatment programs, alcohol/drug programs, self-help groups, and occupational stress injury programs. | | | |
